# Papuan mitochondrial genomes and the settlement of Sahul

**DOI:** 10.1038/s10038-020-0781-3

**Published:** 2020-06-01

**Authors:** Nicole Pedro, Nicolas Brucato, Veronica Fernandes, Mathilde André, Lauri Saag, William Pomat, Céline Besse, Anne Boland, Jean-François Deleuze, Chris Clarkson, Herawati Sudoyo, Mait Metspalu, Mark Stoneking, Murray P. Cox, Matthew Leavesley, Luisa Pereira, François-Xavier Ricaut

**Affiliations:** 1 Instituto de Investigação e Inovação em Saúde, Universidade do Porto (i3S), 4200-135 Porto, Portugal; 2 Instituto de Patologia e Imunologia Molecular, Universidade do Porto (Ipatimup), 4200-465 Porto, Portugal; 3 Laboratoire Évolution and Diversité Biologique (EDB UMR 5174), Université de Toulouse Midi-Pyrénées, CNRS, IRD, UPS, 118 route de Narbonne, Bat 4R1, 31062 Toulouse, France; 4 Institute of Genomics, University of Tartu, 51010 Tartu, Tartumaa Estonia; 5 Papua New Guinea Institute of Medical Research, Goroka, Papua New Guinea; 6 Centre National de Recherche en Génomique Humaine (CNRGH), Institut de Biologie François Jacob, CEA, Université Paris-Saclay, Evry, France; 7 School of Social Science, University of Queensland, Australia, St Lucia, QLD 4072 Australia; 8 Genome Diversity and Diseases Laboratory, Eijkman Institute for Molecular Biology, Jakarta, 10430 Indonesia; 9 Department of Evolutionary Genetics, Max Planck Institute for Evolutionary Anthropology, 04103 Leipzig, Germany; 10 Statistics and Bioinformatics Group, School of Fundamental Sciences, Massey University, Palmerston North, 4442 New Zealand; 11 Strand of Anthropology, Sociology and Archaeology, School of Humanities and Social Sciences, University of Papua New Guinea, PO Box 320, University 134, National Capital District, Papua New Guinea; 12 College of Arts, Society and Education, James Cook University, P.O. Box 6811, Cairns, QLD 4870 Australia; 13 ARC Centre of Excellence for Australian Biodiversity and Heritage, University of Wollongong, Wollongong, NSW 2522 Australia

**Keywords:** Population genetics, Mitochondrial genome

## Abstract

New Guineans represent one of the oldest locally continuous populations outside Africa, harboring among the greatest linguistic and genetic diversity on the planet. Archeological and genetic evidence suggest that their ancestors reached Sahul (present day New Guinea and Australia) by at least 55,000 years ago (kya). However, little is known about this early settlement phase or subsequent dispersal and population structuring over the subsequent period of time. Here we report 379 complete Papuan mitochondrial genomes from across Papua New Guinea, which allow us to reconstruct the phylogenetic and phylogeographic history of northern Sahul. Our results support the arrival of two groups of settlers in Sahul within the same broad time window (50–65 kya), each carrying a different set of maternal lineages and settling Northern and Southern Sahul separately. Strong geographic structure in northern Sahul remains visible today, indicating limited dispersal over time despite major climatic, cultural, and historical changes. However, following a period of isolation lasting nearly 20 ky after initial settlement, environmental changes postdating the Last Glacial Maximum stimulated diversification of mtDNA lineages and greater interactions within and beyond Northern Sahul, to Southern Sahul, Wallacea and beyond. Later, in the Holocene, populations from New Guinea, in contrast to those of Australia, participated in early interactions with incoming Asian populations from Island Southeast Asia and continuing into Oceania.

## Introduction

The island of New Guinea comprises an area of 785,000 km^2^ and hosts around 12 million people (8 million in Papua New Guinea and 4 million in the western Indonesian half of the island), with the highest density in the intermountain valleys 1400–1850 m above sea level (masl). This is one of the most bio-culturally diverse regions on Earth [1] with more than 900 languages spoken, mostly Papuan, but with some Austronesian languages arriving in the last 3 ky [2, 3].

At the time of the initial arrival of modern humans at least 50 kya [4] and substantially earlier [5], New Guinea, Australia, and Tasmania were connected into a single landmass called Sahul, until rising sea levels during the Holocene 9 kya flooded the Torres Strait [6]. In present-day geography, the Pleistocene Sahul continent can be divided into Northern Sahul, representing New Guinea and Near Oceania, while Southern Sahul corresponds to Australia. New Guinea represents approximately a third of the Sahul landmass and the most mountainous part of it, with peaks reaching 4900 masl.

To reach Sahul, the initial settlers from Sunda had to cross up to 90 km stretches of water between the Wallacean islands using still debated southern and/or northern routes via Timor and/or Sulawesi [7–10]. Bradshaw et al. [11] modeled an initial population size of between 1300 and 1550 individuals for the peopling of Sahul, indicating this was a planned crossing involving substantial numbers of people. The eastern part of New Guinea was reached by 49 kya (Ivane valley [12]), the islands of New Ireland by 45 kya, involving a sea crossing of 50 km (Buang Merabak [13]) and the North Solomons by 33 kya with an open sea crossing of 80–180 km depending on the route taken (Kilu cave on Buka island [14]).

New Guineans derive from a biological and cultural mixture of these first Papuan settlers, who arrived around 50 kya [4], and mid-Holocene Austronesian groups closely related to mainland East Asians [2], the latter having the strongest impact on the coast of New Guinea and offshore islands [2, 15–17]. However, early plant domestication and an independent development of agriculture in Highland New Guinea by 9 kya [18] suggest a complex multidirectional exchange of artifacts, plants, animals, and technologies between New Guinea (e.g., banana and sugar cane) and Island Southeast Asia (e.g., pigs and chickens) starting from the mid-Holocene and possibly earlier [19, 20].

Recent genomic data suggest that Indigenous Australians and Papuans diverged from Eurasians 51–72 kya and from each other around 10–32 kya [21]. A later divergence between lowland and highland groups in New Guinea is also attested from the postglacial warming period (10–20 kya) with highland population growth following the spread of plant cultivation around 9 kya [21, 22] and leading to the expansion of Trans-New Guinea languages [23]. Strong genetic differentiation among Papuan groups is observed today, with greater structure for the Y chromosome (paternal lineages) than mitochondrial DNA (maternal lineages), reflecting sex-specific cultural practices [22, 24–26].

These cultural and biological patterns result from the almost 50 kya of Sahul isolation leading to independent genetic and cultural evolution and diversification [21, 23, 27, 28]. New Guineans and Aboriginal Australians can be considered today as the descendants of the earliest modern human group(s) to leave Africa, and are the oldest locally continuous populations found outside Africa. They are also the living groups with the highest traces of archaic introgression from Denisovans (~4%), raising the possibility of a Denisovan presence east of the Wallace line [29], and possible genetic traces of a very early and elsewhere extinct expansion of modern humans out of Africa [27].

However, despite this exceptional context of New Guinean population history, fewer than 20 Pleistocene sites are known for all of New Guinea [30] and there are very limited genetic data available, compared with other regions of the world. Our understanding of the population dynamics that led to the current situation in New Guinea is therefore still largely unknown.

Here we report the largest mitogenome dataset for New Guinea, including 379 new genomes, and investigate four key points about the scenario of human arrival in Sahul that are still unclear and contentious: (1) the number of different groups of settlers involved, (2) the date of first human arrival (65 kya and/or 50 kya; [4, 5]), (3) the route(s) taken by the first settlers (northern and/or southern routes) [7–10], and (4) the nature of population substructure following the peopling of Sahul [22, 25, 26, 31–33].

## Material and methods

### Sampling and mtDNA sequence generation

The samples analyzed in this study were drawn from populations across Island South East Asia, Oceania and Australia. The dataset of 915 complete mtDNA genomes (Tables S1, S2, and S3) includes mtDNA sequences compiled from (1) newly collected samples from Papua New Guinea, (2) archival biological samples from the Institute of Medical Research (IMR) of Papua New Guinea, and (3) previously published studies.

A total of 123 DNA samples were collected during 2016 and 2017 field seasons in Papua New Guinea, and cover individuals from all 22 Papua New Guinea provinces (Table S1). All samples were collected from healthy unrelated adult donors after informed consent forms were signed. In each sampling location, a full presentation of the project was made, followed by discussion with each donor to ensure that they fully understood the project. Participants were surveyed for language affiliation(s), current residence, date and place of birth, and a short genealogy up to three or four generations to establish regional ancestry. Saliva samples were collected using the Oragene DNA Collection Kit (DNA Genotek Inc., Ottawa, Canada). DNA was extracted according to the manufacturer’s instructions.

A further 256 samples were selected from the IMR archival biobank, derived from blood samples collected in the 1980s, with new ethics approvals obtained in 2015. These samples cover four highland provinces and one coastal province (Table S1). DNA was extracted using the DNA Blood Mini Kit (QIAGEN, Hilden, Germany) and whole-genome amplified with the Illustra GenomiPhi V2 kit (GE Healthcare, Chicago, IL, USA) following the manufacturer’s instructions.

Complete mitochondrial DNA sequences were generated for all 379 new and archival samples using two approaches. For 73 newly collected samples and 256 IMR samples, complete mtDNA sequences were generated following the protocol described in Brucato et al. [34]. Briefly, double bar-coded libraries were prepared and enriched for mtDNA, as described previously [35, 36]. For 50 newly collected samples, complete mtDNA sequences were extracted from whole genome sequencing performed at CNRGH, France (Table S1). Sequencing libraries were prepared using TruSeq DNA PCR-Free and TruSeq Nano DNA HT kits depending on DNA quantity. 150 bp paired-end sequencing was performed on the Illumina HiSeq X5 System (CNRGH). For all samples, consensus sequences were obtained after base-calling, quality filtering, and further quality control steps to obtain consensus sequences, as described previously [37]. The 379 new complete mtDNA sequences have been deposited in GenBank (http://www.ncbi.nlm.nih.gov/genbank/) under accession numbers MN849490–MN849868.

A comparative dataset of 539 individuals was built by compiling all of the published complete mtDNA sequences affiliated with haplogroup P, Q, and M (M27, M28, M29) described previously for people of Papuan ancestry (Table S4). These sequences were identified by screening the main web-based mtDNA databases: the DDBJ/EMBL/GenBank international nucleotide sequence database, Phylotree [38], and Family Tree DNA (https://www.familytreedna.com/). The final dataset includes 915 mitogenomes from haplogroup P, Q, and M, including the 379 new and archival sequences from Papua New Guinea generated in this study (Tables S1–S3).

All new, archival, and comparative sequences were analyzed and aligned against the revised Cambridge Reference Sequence [39] using the MAFFT aligner v.7 [40]. Mitochondrial haplogroups were determined using Haplogrep [41] based on PhyloTree Build 17 [38].

### Mitochondrial DNA analysis

Phylogenetic relationships were analyzed by constructing maximum parsimony trees using the whole mtDNA sequences affiliated with haplogroups of Papuan ancestry M27 (*n* = 144 samples), M28 (*n* = 73), M29’Q (*n* = 361), and P (*n* = 302) (Figs. S1–S4), guided by published principles [38].

To estimate the time to the most recent common ancestor (TMRCA) of the clades, the maximum parsimony trees were used for maximum likelihood (ML) estimations (Table S4), by considering two widely used mutation rates so that our estimates are comparable with published ones: Fu et al. (mean = 2.67 × 10^−8^ substitutions per site per year, SD = 2.6 × 10^−9^) and Soares et al. [42] (mean = 1.67 × 10^−8^ substitutions per site per year). These authors extensively evaluated the effect of demographic effects, and, in the latter case, also of selection effects, on their estimations. It was not our aim to add another mutation rate to the ones already available and extensively used. The Soares clock accommodates the effects of purifying selection, tending to attribute older ages that will function as the maximum limit to our estimates. The mutation rate from Fu et al. [43] was calibrated on radiocarbon dated ancient sequences, and the used method provides inferences that minimize the effects of rate temporal dependency, and will work here as the minimum limit to our estimates. We performed the ML estimates of branch lengths using PAML v.4 [44], assuming the HKY85 mutation model with gamma-distributed rates, excluding indels and hotspot mutations, as reported previously [38].

**Fig. 1:**
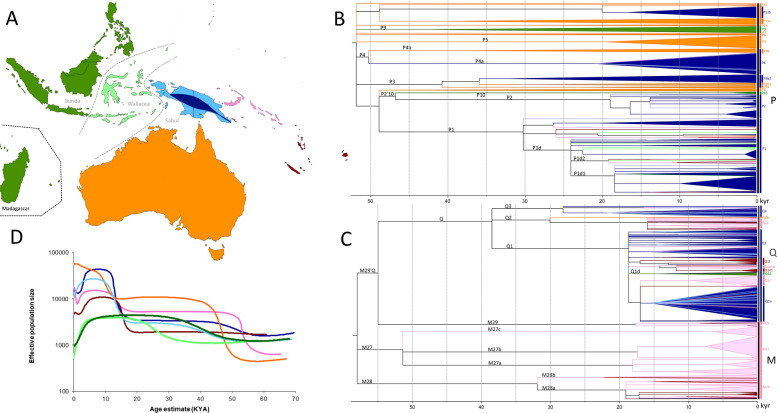
Genetic relationships and increasing population sizes across Sunda and Sahul. **a** Seven geographical regions: coastal PNG (light blue), highland PNG (dark blue), Near Oceania (pink), Remote Oceania (red), Australia (Orange), Wallacea (light green), and Sunda (dark green). **b** Tree of haplogroup P. Subclades are represented by triangles, while single lineages are represented by lines. The tree is scaled to kya (thousands of years ago) using the maximum likelihood molecular clock for the whole mtDNA genome with the mutation rate of Fu et al. (details of age estimates are reported in Table S1). **c** Tree of haplogroups M and Q. **d** Bayesian skyline plot representing median estimates of effective population size for each the seven geographic regions based on P, M, and Q mtDNA lineages

The timing of modern human arrival in Northern Sahul, based on the mtDNA genomes analyzed in this study, was estimated from the TMRCA of the main Papuan haplogroups using both the Fu and Soares mutation rates. While confidence intervals estimated with either mutation rate tend to overlap, we favor Fu et al. [43] age estimates because they are based on directly dated fossils that are within the time range of modern human evolution considered here. However, older age estimates given by the Soares mutation rate cannot be ruled out. Thus, skeletal representations of the tree (Fig. 1) were drawn using FigTree (tree.bio.ed.ac.uk/software/figtree/), with the tree scaled using the ML TMRCA estimates using Fu et al.’s [43] mutation rate.

The spatial frequency distribution of the main Papuan haplogroups was estimated with the ESRI ArcGIS software package (www.esri.com/software/arcview/), based on the frequency data for each haplogroup and the latitude and longitude of the center points for each of the seven regions considered in this study (Near Oceania, Remote Oceania, Coastal New Guinea, Highland New Guinea, Australia, Wallacea, and Sunda) (Table S5). The frequency maps were created using the “Inverse Distance Weighted” (IDW) option, with a power value of two for the interpolation of the surface. IDW assumes that each input point has a local influence that decreases with distance based on the assumption that samples close to one another should be more alike than those that are farther apart. However, as it accounts only for the effects of distance, the color scale in regions with few or no samples (e.g., Australia for haplogroups M28/27/29, Indonesia for haplogroup P and Q) should not be considered as having high statistical confidence. Details regarding the different haplogroup frequencies in these regions should be verified in Table S5.

To assess effective population changes through time for Papuan haplogroups (M27, M28, M29’Q, P), Bayesian Skyline Plots (BSPs) were calculated using BEAST v.1.8 [45] and visualized with Tracer v. 1.6 (http://beast.bio.ed.ac.uk/Tracer). A 25-year generation time was assumed [46]. BSPs estimate the effective population size through time using random sequences from a given population but have also proved effective with individual haplogroup data. For this analysis, we used Fu’s mutation rate. BEAST uses a Markov chain Monte Carlo (MCMC) approach to sample from the posterior distributions of model parameters (branching times in the tree and substitution rates). Specifically, we ran 100,000,000 iterations, with samples drawn every 10,000 MCMC steps, after a discarded burn-in of 10,000,000 steps. We checked for convergence to the stationary distribution and sufficient sampling by inspection of posterior samples.

## Results

### Haplogroup affiliation in Northern Sahul

Of the 379 new complete mtDNA genomes, 28 individuals (7.4%) are affiliated to nonindigenous Northern Sahul haplogroups B4a1a1 (*n* = 25) and E1a (*n* = 3) and their descendant lineages. These haplogroups are only detected in the coastal region of New Guinea and the Bismarck Archipelago (Table S1 [47, 48]), while their ancestral lineages are thought to have entered the New Guinea region from mainland Asia following postglacial and/or Austronesian expansions in the early and mid-Holocene, respectively [48–50]. Overall, their current distribution demonstrates limited penetration inland into New Guinea itself in agreement with previous studies [47, 48, 51–53].

The remaining 351 mtDNA genomes include two minor indigenous haplogroups from Northern Sahul, R14 and M73 [54, 55]. Each present in only one highland PNG individual, they are too rare to be informative in the frame of this study and were not analyzed further.

The vast majority of the mtDNA genomes, 349 (92% of 379), are affiliated with the main indigenous Northern Sahul haplogroups—M27 (1.3%), M28 (<1%), M29 (<1%), Q (47%), and P (43%). Considering the entire dataset (Table S5), these indigenous lineages represent 1818 (57%) individuals in Northern Sahul (M27 8.2%, M28 8.8%, M29 1.5%, Q 25%, and P 13.5%); a number that rises to 2200 when all regions worldwide are considered. These indigenous Northern Sahul haplogroups are the focus of this study.

### Northern Sahul phylogeography

**Fig. 2:**
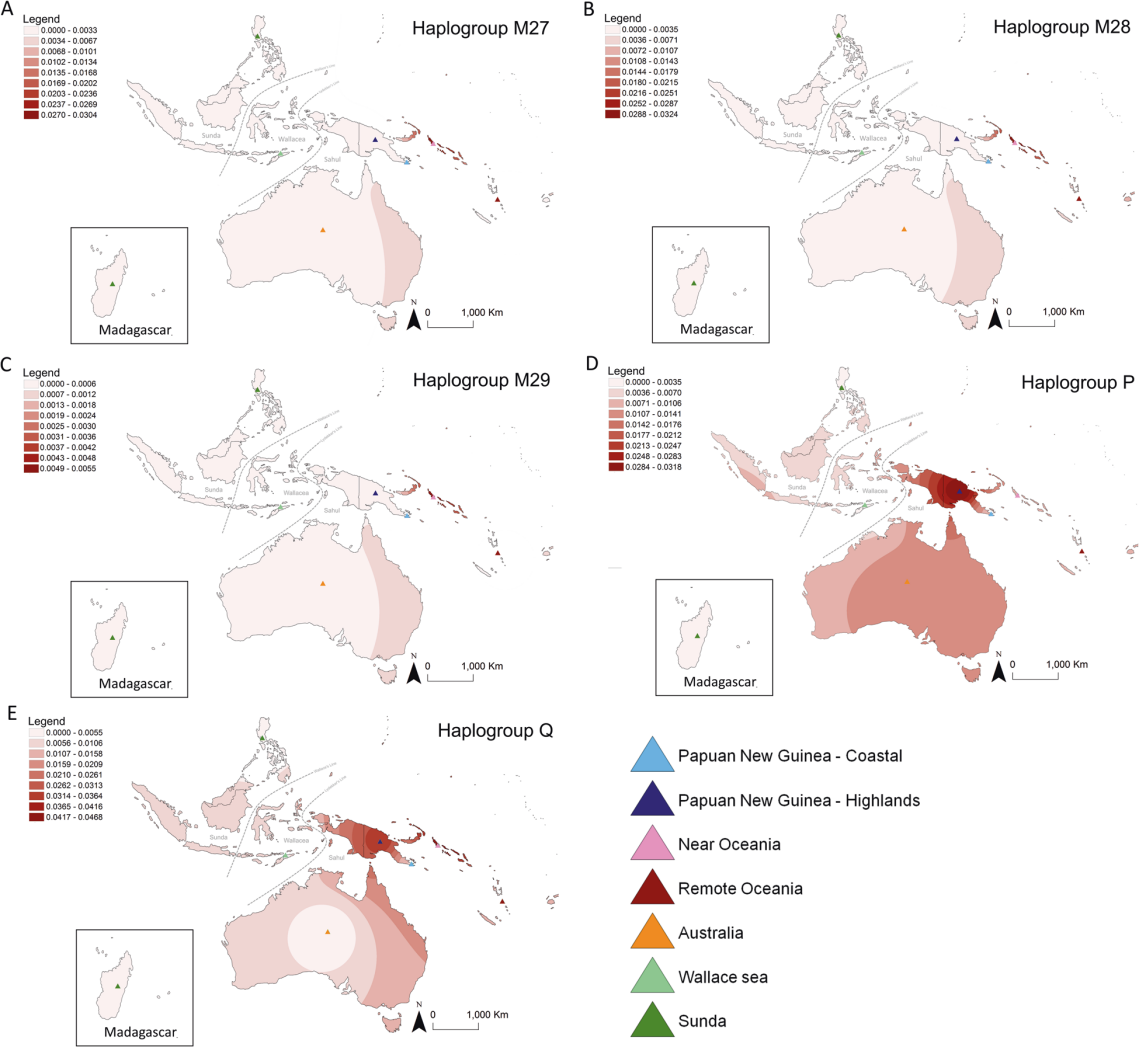
Spatial distribution of main Sahul mtDNA haplogroups. Inverse distance weighted interpolation shows areas with higher frequencies in darker shading, taking into account only the effects of distance (color scale in regions with few or no samples should not be considered as having high statistical confidence). Data details are provided in Table S5 and the triangles represent the central point for each region used in the interpolation. **a** Distribution of haplogroup M27. **b** Haplogroup M28. **c** Haplogroup M29. **d** Haplogroup P. **e** Haplogroup Q

The geographic distribution of the major indigenous haplogroups from northern Sahul (M27, M28, M29, Q, and P) reveal strong geographic patterns for each of the five haplogroups (Fig. 2a–e), centered on northern Sahul, where they harbor—except for some P subhaplogroups—their greatest frequency and diversity (Figs. 1 and S1–S4). However, when we look in more detail at the geographic distribution of their subclades, associated phylogenetic trees and coalescence age estimates (Table S4, Figs. 1 and S1–S4), we can identify three main groups of indigenous Northern Sahul lineages.

The first group includes haplogroups M27, M28, and M29, which are almost exclusively found in Near Oceania (e.g., the Bismarck and Solomon archipelagos), and may have originated in this region as they are most diverse and frequent here (Tables S1, S2, S5 and Fig. 2a–c; [47, 48, 52, 56]). These haplogroups are absent from Australia, and are rare (<1%) in the highlands of Papua New Guinea and in Remote Oceania (Table S5), whose lineages root in haplotypes found only in the Bismarck and Solomon archipelago. From their age estimates (Table S4), haplogroups M27, M28, and M29 found outside Near Oceania appear to be related to increasing population interactions within the region during the mid-Holocene.

Coalescence ages estimated for M29’Q ~55 kya (95% CI 42–67 kya) and M27 ~51 kya (95% CI 40–62 kya) are in broad agreement with dates for the first settlement of Sahul from archeological and genetic data [4]. At ~32 kya (95% CI 22–42 kya), the M28 age estimate is close of the beginning of the Last Glacial Maximum (28 kya). Long branches on the phylogenetic tree for haplogroups M29, M27a,b,c, and M28 may suggest long-term isolation without expansion for these lineages after their initial arrival in the Bismarck and Solomon archipelagos (Fig. 1), while other explanations for this pattern—unsampled diversity or lineage disappearances—cannot be fully ruled out.

The next main lineage diversifications took place around the transition period between the Last Glacial Maximum (28–18 kya) and the postglacial warming period (18–10 kya) [18, 57] (M27a: 18 kya, M27b: 17 kya, M28a: 19 kya, M28b: 24 kya, M29: 18 kya) and extended into the Holocene period of increasing population interaction (Figs. S1–S3 and Table S4). All derived lineages arising from these two expansions periods are clustered geographically, suggesting limited dispersal over time despite major climatic and historical changes.

The second group includes haplogroup Q and its subhaplogroups Q1, Q2, and Q3, all of which have their greatest frequency and diversity in Northern Sahul (highland and coastal New Guinea and Near Oceania) (Figs. 1, S3, and  2e; [48, 49]), suggesting probable origins within this region. Phylogenetic analysis indicates that the Q branch diverged from M29’Q around ~55 kya (95% CI 42–67 kya), and that after a period of isolation of nearly 15 kya (long branch, Fig. 1), the Q haplogroup diversified into three subhaplogroups around 38 kya (95% CI 28–52 kya), a time between initial settlement and the Last Glacial Maximum (28–18 kya). Haplogroup Q2 in Near Oceania and Q3 in highland New Guinea show diversification early in the Last Glacial Maximum period, ~30 kya (95% CI 20–40 kya) and 28 kya (95% CI 20–36 kya) respectively, while Q1 in Highland and coastal New Guinea and Near Oceania diversified at the end of Last Glacial Maximum ~19 kya (95% CI 15–22 kya). As seen for M27, M28, and M29, derived lineages of these subclades have tended to stay in the same geographical region with little evidence of spreading across Northern Sahul.

Haplogroup Q1, with a coalescence age estimate of ~18 kya, shows geographic clustering, with highest frequency and diversity in highland New Guinea (Q1a, Q1f, and >10 related but unnamed subclades) and Near Oceania (Q1b, Q1c, and Q1e). Few lineages rooted in these subhaplogroups are detected outside Northern Sahul. These are found in the Sunda and Wallacea islands (Q1d, also Taiwan, Philippines, and Madagascar), in northern Australia (Q1a), and in Remote Oceania (Q1b, Q1e, and Q1f subclades in the Solomon Islands, Vanuatu, Fiji, Samoa, and the Cook Islands). All have coalescence ages within the postglacial warming period (18–10 kya) or following the Holocene. The three Q1 lineages present in Australia were all identified in individuals with Torres Strait Islander ancestry, which are known for their close links with New Guinea [32], and are associated with the Q1 subclade expansion during the postglacial warming period (18–10 kya) (Fig. S3).

Haplogroup Q2 has a coalescence age estimate at ~30 kya, and is also strongly geographically structured, with greatest diversity and frequency in Near Oceania, particularly the Bismarck Archipelago. This is in agreement with proposed demographic expansions following the initial settlement of New Britain around 35 kya [58]. Diversification of this haplogroup began in the post-Last Glacial Maximum period and continue into the Holocene, but was largely restricted to Near Oceania. However, a few lineages have been observed in Remote Oceania with coalescence ages in the late Holocene (Fig. S3 and Table S4). We confirm the presence of Q2b in one western Indigenous Australian. This mtDNA haplotype branches deeply within the Q clade [54] and may reflect earlier connections (predating the Last Glacial Maximum, Fig. S3) between Northern and Southern Sahul populations.

Haplogroup Q3 shows a similar pattern, with a distribution center in the highlands of New Guinea, where it has its higher diversity and a coalescence age of ~28 kya (early in the Last Glacial Maximum period). However, several Q3a and Q3b subclades have been detected in coastal New Guinea (Q3–215 ~21 kya, preQ3b ~16 kya, Q3b ~7 kya), Near Oceania (preQ3b ~16 kya) and Timor (Q3–215 ~21 kya), all associated with coalescence ages from the end of the Last Glacial Maximum (28–18 kya), postglacial warming or Holocene periods (Table S4 and Fig. S3). Of particular note, the Q3 lineages shared between Timor and coastal New Guinea are deep branching and have a coalescence age of ~21 kya (Table S4 and Fig. S3) within the late Last Glacial Maximum period, reflecting a possible ancient connection between the island zone around Timor and continental Northern Sahul [55].

The third group includes haplogroup P, with a coalescence age of ~51 kya (95% CI 44–60 kya), and its numerous subhaplogroups (P1–P12) (Phylotree.org). Haplogroup P has clades with deep branches rooted in the basal P clade, distributed across Southern Sahul (P3a, P3b2, P4b, P5, P6, P7, P8, new P13a (P-153G, Fig. S4)) and Northern Sahul (P1, P2, P3b1, P4a, new P13b1). This lineage has also been identified outside Sahul in the Philippines (P9 and P10 in the Aeta and Agta indigenous groups), suggesting that the P haplogroup may have evolved in Sunda or Wallacea (Fig. 1, Table S4, and Fig. S4) [55].

Both Northern and Southern Sahul P haplogroups have old coalescence ages (around 50–45 kya, 95% CI 32–62 kya) (Table S4) some probably related to the early settlement period P4 ~50 kya (95% CI 42–58 kya), P6 ~50 kya (95% CI 41–58 kya), Pre-P1 ~49 kya (P-16176T) (95% CI 38–60 kya), P-13: ~49 kya (95% CI 34–64 kya), pre-P8 ~48 kya Pre-P1 (95% CI 33–62 kya), P2’P10 ~47 kya (95% CI 35–58 kya), P9 ~43 kya (95% CI 30–55 kya), P3 ~41 kya (95% CI 32–50 kya). In general, P haplogroups diversified earlier in Southern Sahul (older coalescence age) than in Northern Sahul, which occurred from the end of the Last Glacial Maximum through to the postglacial and Holocene periods, followed by long-term isolation (long branches on the tree) (Table S4 and Fig. 1). While some rare sharing of P lineages within and outside Sahul suggest some level of population interaction (discussed below), most derived lineages are geographically clustered (Figs. 1 and S4; for Southern Sahul haplogroups, see [31–33, 54]).

Interestingly, haplogroup P includes some subhaplogroups restricted to Southern or Northern Sahul, but distantly connected as diverging from the same root P haplogroups (Fig. 1, Table S4, and Fig. S4). Coalescence ages and phylogenetic reconstructions suggest two unexpected pattern for these haplogroups, due either to the sharing of an ancestral population or a signature of ancient population connections between Southern and Northern Sahul.

On one hand, Northern Sahul hosts specific P haplogroups such P1, P2, P4a, and P13b, which are both more frequent and diverse in highland New Guinea, supporting their possible emergence/diversification in this region. These lineages have coalescence ages dating back to the early settlement phase, before 45 kya (Fig. 1, Table S4, and Fig. S4). These ancient P haplogroups specific to northern Sahul (P1, P2, P4a, and P13b) diverged from their sister Southern Sahul haplogroups within a very restricted time window (50–45 kya). Indeed, we observed that P1, P2, P10, and pre-P8 are related (Figs. 1 and S4). They diverged around 49 kya (95% CI 38–60 kya), in separate clades that today are geographically isolated: P10 in Wallacea, P2 and P1 in Northern Sahul, and pre-P8 in Southern Sahul. A similar pattern of an early split is observed for P4: ~50 kya (95% CI 42–58 kya) between the Northern (P4a) and Southern (P4b) Sahul subhaplogroups; and for P-13: ~49 kya (95% CI 34–64 kya) between the Northern (P13b) and Southern (P13a) subhaplogroups. This pattern suggests Northern and Southern Sahul populations shared an ancestral population that harbored high P diversity.

On the other hand, P1 haplogroups, the most frequent and diverse P haplogroup in Northern Sahul, are dominated by lineages diverged from highland New Guinea P1 haplogroups during the Last Glacial Maximum (18–28 kya) and spread geographically to other regions during the Last Glacial Maximum and postglacial periods: coastal New Guinea (Madang, P1d2 19 kya, P1d1 18 kya, and P1d5 16 kya), Near Oceania (Solomon Islands, P1g 26 kya, P1–152C 26 kya, P1d2 19 kya, and P1d1a 18 kya), Remote Oceania (Tonga, Fiji, Polynesia, P1d2 19 kya), Wallacea (Timor, P1–152C 26 kya, P1d 24 kya, P1d3 23 kya, P1d2 19 kya and Maranao in the Philippines, and P1d1c 17 kya) and Australia (P13b2 25 kya, P1–152C 26 kya; Torres strait ancestry individuals, Nagle et al.). Similarly Australian carriers of P3b1 have a Northern Sahul origin, supported by their Torres Strait ancestry and clustering with highland New Guinea P3b2 [31].

### Demographic expansions

These settlement and expansion dates are corroborated by Bayesian skyline estimates obtained for each of the seven geographical regions when focusing only on P, M, and Q diversity (Fig. 1d). Near Oceania has the first population increase, starting soon after initial settlement (55 kya), while Australia displays a substantial population increase at a slightly later time but still in the same time frame. Coastal and highland New Guinea populations show smaller population increases at the same time as Near Oceania. We note that BSPs for Australia only include haplogroups shared with Northern Sahul (P haplogroups), which represents just a third of Indigenous Australian diversity [31–33]. However, this still represents a reasonable proxy for Australian diversity as all Australian lineages (O, P, S, and M) seem to show the same general process of expansion beginning ~50 kya following initial settlement of Australia [31–33].

All Sahul regions show population expansions in the postglacial warming period (18–10 kya), including within those lineages that later contributed to the initial settlement of Remote Oceania. Notice that Bayesian skyline estimates for Sunda and Wallacea are less reliable as these regions have many other haplogroups than those focused on here, and thus cannot be readily compared with inferences for northern Sahul where P, M, and Q comprise almost 100% of the local diversity.

## Discussion

### First phase of Sahul settlement

This study shows that the mtDNA diversity of Northern Sahul does not originate from Southern Sahul. This is supported mainly by the fact that (1) mtDNA lineages found in New Guinea are not derived lineages of those found in Southern Sahul (Figs. 1 and S4), (2) the two regions of Northern and Southern Sahul host different deep rooted lineages with strong geographic structuring (Northern Sahul: M27, M28, M29, Q2, P1, P2, P10, P4a, and P13b; Southern Sahul: O, S, N13, M42a’c, P5, P6, P7, P8, and other P), and (3) all of these haplogroups are rooted in the age range of the initial settlement phase of Sahul (>50 kya), supported by most archeological and genetic evidence [4, 5, 30]. A similar rationale can be used to support the view that Southern Sahul mtDNA diversity is not derived from Northern Sahul diversity (Figs. 1 and S4 [31–33]).

The different origins of the Northern and Southern Sahul mtDNA profiles could be explained by two main hypothesis. Either (1) ancestral Sahul settlers carried all of the major haplogroups, and during their rapid dispersal within Sahul, carriers of specific haplogroup settled different regions, or (2) two (or more) groups of settlers carrying different haplogroup sets both reached Sahul during the early settlement phase (>50 kya), with one group settling Southern Sahul and the other group Northern Sahul.

The most parsimonious explanation, when considering the phylogeographic, phylogenetic, and coalescence age results, favors the second hypothesis: two (or more) groups of settlers likely originating from a common ancestral Sunda population. Indeed, (1) it is unlikely that a single group of settlers carrying all ancestral haplogroups could alone explain the strong geographic structure observed today—with two different profiles of ancestral lineages in Southern and Northern Sahul not derived from each other—either by a peculiar settlement pattern or genetic drift. (All haplogroups found in Northern Sahul would have to have been lost in Southern Sahul, and vice versa). (2) There are differences in age estimates of some of the Northern and Southern haplogroups suggesting different timings of arrival for various haplogroups. (3) Today, the oldest Sahul haplogroups M29’Q (~55 kya, 95% CI 42–67 kya) and M27 (~51 kya, 95% CI 40–62 kya) are restricted to the eastern part of Northern Sahul (Table S4 [47]). This is in line with different demographic expansion times in Northern and Southern Sahul, possibly related to at least two separate dispersal events (Fig. 1d). This finding is consistent with recent migration modeling suggesting entry points into both northern and southern Sahul were likely [7, 9].

Regarding the ancestral populations of Sahul, nuclear data support a common ancestral population of Northern and Southern Sahul settlers located in Sunda [21]. Strong population structure within this Sunda population can be postulated based on mtDNA diversity patterns (e.g., P haplogroup diversification with deep rooted P lineages found in Sunda, Northern, and Southern Sahul). Autosomal data is therefore more in agreement with the hypothesis of two groups of Sahul settlers carrying different mtDNA lineages.

Regarding the routes used by the first settlers, our results do not allow us to disentangle the use of a northern (via Sulawesi and the Bird’s Head of New Guinea) or southern (via Timor and the northwest shelf of Australia) routes into Sahul, which from paleogeographic, environmental, and demographic reconstructions are both possible [7–10]. However, in the event of the arrival of two Sahul settler groups, the one leading to the settlement of Northern Sahul ending in the Bismarck archipelago may favor an origin within the maritime and coastal adapted cultures of Wallacea, and thus the use of the northern coastline route [10]. Similarly, the group leading to the settlement of Southern Sahul may favor the southern route across the savanna corridor running from Sunda (the Java plain) to Sahul (the Arafura plain) [59]. The use of both routes is finding some support in archeological evidence, with sites >40 kya old located along both paths [30]. However, lineages shared between Northern Sahul and Australia (Q2b, 30 kya), Timor (Q3–215, 21 kya) and Sunda (Q1d 14 kya) suggest that a southern route of interaction was active at a later period, possibly reflecting the more ancient settlement path [7].

Regarding the timing of the initial settlement of Sahul, while old age estimates of lineages agree with settlement over a rather narrow time window (55–45 kya), the detailed scenario is still unclear. The suggested 65 kya human occupation of Madjebebe (Northwestern Australia [5]) sits within the range of confidence intervals for some of the oldest Sahul haplogroups (M29’Q: 42–67 kya, M27: 40–62 kya) regardless of the mutation rate used (Table S4 and Fig. 1). On one hand, some of our results favor an early arrival of Near Oceania ancestors, based on the older age estimate of some northern Sahul haplogroups (M27, 51kya; M29’Q, and 55kya; Table S4) and early Near Oceania demographic expansions (Fig. 1d). On the other hand, the phylogeny of the P1, P2’P10, and pre-P8 cluster suggest that Southern Sahul haplogroup emerged earlier (pre-P8, 48kya) than Northern Sahul and Sunda related haplogroups (P2’10, 47kya). However, several potential biases—a limited number of southern haplogroups used for the Bayesian skyline analysis, demographic expansion occurring in Sunda rather than in Sahul, lineages lost in the last 50 kya and blurring the original demographic signal—do not allow clearer genetic timings.

The scenario we might postulate from these results suggests (1) the diversification of ancestral Sahul lineages in a population located in Sunda, (2) two (or more) dispersal events to Sahul within the same narrow time frame around 45–55 kya, (3) a dispersal to Sahul of a group of settlers carrying lineages observed today in Northern Sahul (M27, M28, M29’Q, P1, P2, P4a, P10, P13b), (4) another dispersal to Sahul of a group of settlers carrying the lineages observed today in Southern Sahul (O, S, N13, M42a’c, P5, P6, P7, P8, and other P), and (5) an absence of detectable interactions (such as shared lineages and admixture) between Northern and Southern Sahul groups during the initial settlement phase and the following 20 ky years, until contacts increased after the Last Glacial Maximum.

### Regionalism and isolation in Northern Sahul

Phylogeographic and phylogenetic analyses of northern Sahul haplogroups show strong geographic clustering of the different haplogroups and their derived lineages, suggesting that populations were structured early after the initial settlement of Northern Sahul (Figs. 1 and S1–S5). This is in good agreement with the nomadic sedentism and strong territoriality observed today in New Guinea societies [23, 60]. It also mirrors the “regionalism” observed in the mtDNA lineages of Indigenous Australians [33], suggesting that Northern and Southern Sahul populations exhibited similar regionalism patterns.

The main differences between Northern and Southern Sahul reflect the date at which this strong geographic structure appeared. Southern Sahul haplogroups (e.g., P, O, S, and M42a’c) show rapid diversification following initial arrival in their new environment, and then to a large extent, geographic stability with few large scale movements over the last 55 ky (Figs. 1 and S4 [33]). In Northern Sahul, haplogroups show more diverse patterns. In Near Oceania, haplogroups M27, M28, and M29 exhibit a geographic distribution restricted to a small geographical region over the last 55 ky, similar to that observed in Southern Sahul. On New Guinea, however, haplogroups Q and P and their derived lineages also have a restricted geographic distribution, but (1) this structure arose more recently in the Last Glacial Maximum, postglacial and Holocene periods, and (2) the structure resulted from both local lineage diversification and the arrival of new lineages from external regions, indicating some degree of movement within and beyond Sahul from the mid-Pleistocene onwards (Figs. 1 and S3, S4).

Another difference between Southern and Northern Sahul is the period of lineage isolation (longer branches on the tree with greater accumulation of mutations) before diversification. Following the initial settlement of Northern Sahul by 50 kya (Ivane valley 49 kya, Buang Merabak 45 kya [30]), we observe a pause of around 20 ky before most Northern Sahul lineages diversified (Figs. 1 and S1–S4). This suggests an unusual pattern: human occupation of a new territory without a concomitant genetic signal of demographic expansion. Diversification instead first occurred during the Last Glacial Maximum period (M28 32 kya, Q1 18 kya, Q2 30 kya, Q3 28 kya, P1 30 kya, and P2 19 kya), and may have been triggered by environmental changes such as the landmass increase during the Last Glacial Maximum (28–18 kya [57, 59]). This later diversification period is also confirmed by distantly connected P subhaplogroups, which show later diversification in New Guinea (P4a 21 kya, P13b 19 kya) compared with their sister haplogroups in Southern Sahul (P4b 38 kya, P13a 40 kya), although all are present from the initial settlement period.

The reasons behind this observed longer isolation (Figs. 1 and  S1–S4) and later diversification (Table S4) of many Northern Sahul haplogroups has still to be clarified, and is surprising considering the rapid adaptation of the first settlers to the new fauna, flora, and environments (tropical rainforests, semiarid plains, and upper mountain grasslands) encountered in New Guinea and Australia [30]. However, a combination of factors may have delayed population expansion in New Guinea itself—a harsh environment in northern mountainous Sahul, and greater shellfish coastal resources, competition with other occupants (e.g., megafauna or other members of the *Homo* genus)—or signals from this early period may have been lost in the current mtDNA gene pool.

### Dispersal episodes from Northern Sahul

Northern and Southern Sahul lineages underwent a period of diversification immediately following initial settlement, and then subsequently during the Last Glacial Maximum, postglacial and Holocene periods (Figs. 1 and S1–S4). Some of the derived lineages resulting from these events are found outside their region of origin, and provide clues to ancient population interactions. This case is even more informative for Northern Sahul. While Southern Sahul haplogroups have very limited geographic ranges within Australia (expansion of the O2 derived lineage in the Holocene, and re-expansion in the Western central desert in postglacial period around 15 kya) [33], the Northern Sahul lineages show a much wider geographic range of expansion, also reaching Southern Sahul, the far east (Remote Oceania) and the far west (Sunda).

Northern Sahul hosts haplogroups that have locally diversified within New Guinea (P1 Q1, Q3) and Near Oceania (M27, M28, M29, and Q2), and from which a limited number of derived lineages have spread in neighboring regions (Figs. S1–S4 and 3).

**Fig. 3:**
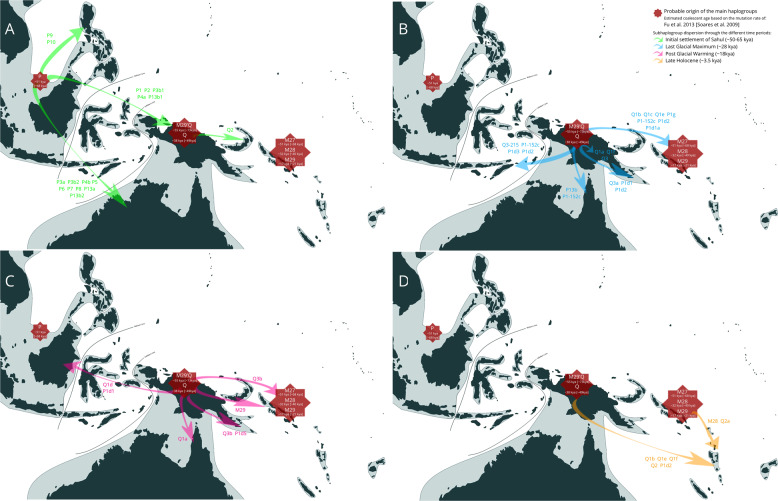
Proposed movements of maternal lineages P, M, and Q. Dark shading represents modern coastlines; light shading illustrates the extent of the Sunda continent at the Last Glacial Maximum. Red octagrams represent the probable approximate origins of haplogroups. Arrows represent probable migration paths during: **a** the initial settlement of Sahul (~50 kya; green); **b** the Last Glacial Maximum (~28 kya; blue); **c** the postglacial warming period through to the Holocene (~18 kya; pink); and **d** the Late Holocene (~3.5 kya; orange)

First, our data support population interactions in both directions between New Guinea and Near Oceania, starting from the end of the Last Glacial Maximum and continuing into the postglacial and Holocene periods. New Guinea lineages are found in the neighboring region of Near Oceania, including preQ3b (16 kya), Q1b,c,e (14–5 kya), and several P1 subhaplogroups (26–18 kya). Lineage P1d2 was later carried into Remote Oceania during late Holocene. Near Oceania lineages spread within the same periods into New Guinea (M28a8 17 kya, Q2a4 12 kya, M29a,b 9–2 kya) and later to Remote Oceania (M29a,b around 2 kya). This is in agreement with archeological evidence, which supports some level of connection between New Guinea and Near Oceania from the end of the Last Glacial Maximum (around 23 kya) based on animal, plant, and object (e.g., obsidian) translocation [23]. Interestingly, we did not detect genetic interactions for the earlier period lasting from the first settlement of Near Oceania 45 kya (Buang Merabak [13]) to the end of Last Glacial Maximum (18–30 kya). This may suggest that after being reached 45 kya by crossing the Vitiaz Strait from mainland New Guinea, this region of Near Oceania remained largely isolated from the rest of Sahul for more than 20 ky, despite the absence of known barriers that humans had already overcome to reach Near Oceania in the first place [61].

Second, our results also attest long-term interaction between Northern Sahul and Northern Australia from early in the Last Glacial Maximum (30 kya) to postglacial periods (18–10 kya), but in a unidirectional manner (from Northern to Southern Sahul). Indeed, haplogroups from New Guinea (P3b 36 kya, P1–152c 26 kya, P13b 20 kya, Q1a 15 kya) and Near Oceania (Q2b 30 kya) are present in Northern Australia. This result broadly matches those obtained from Y chromosome [26] and nuclear [21] data, although the Y chromosome supports a much more recent split (9–12 kya) between Papuans and Indigenous Australians than nuclear DNA (10–32 kya [21]). Our mtDNA results indicate that interaction between New Guinea and southern Sahul stopped before the Holocene and before the geographical separation between Australia and New Guinea (6–8 kya) [6].

Third, Northern Sahul haplogroups from New Guinea are detected in Wallacea from the Last Glacial Maximum and postglacial warming periods onward (Timor Q3–215 21 kya, P1 subhaplogroups 19–26 kya), as well as on major regional islands (Q1d 14 kya in Taiwan and Q1d and P1d1c 17 kya in Philippines). Lineages moving during the last 30 kya from Northern Sahul to the Wallacea and Sunda regions would have been mediated by maritime interactions, in agreement with the “voyaging corridor” hypothesis and an increase in maritime interactions between Northern Sahul and Island Southeast Asia from the end of the Pleistocene [50, 62].

## Conclusions

To summarize, our results suggest that lineage dispersals from Northern Sahul likely result from environmental changes related to the Last Glacial Maximum and postglacial periods. In the former period, colder, and dryer conditions in Northern Sahul led to increases in the Sahul landmass and may have motivated population movements within and outside this region without strong associated demographic expansions (Fig. 1d). Consistent with the limited range of lineages involved, this may reflect refugial movements (Figs. 1 and S1–S4). The more favorable conditions of the postglacial warming period (10–18kya) led to demographic expansion and accentuated lineage diversification (Fig. 1d), leading to the geographic dispersal of Northern Sahul lineages within and outside Sahul. Rising sea level during the Holocene saw an intensification of this pattern with geographically restricted demographic expansions, and ultimately, Pacific settlement from Near Oceania.

The maternal history of populations from Northern Sahul, one of the oldest continuous populations outside Africa, thus sheds light on the population history of this region. This study proposes an initial arrival to Sahul of two groups of settlers within the same broad time window (50–65 kya), each carrying a different set of maternal lineages, with one group settling Northern Sahul (New Guinea and Near Oceania), and one Southern Sahul (Australia). Following a period of a least 20 ky of relative isolation of Northern Sahul population, the cause of which is still unclear, the postglacial period after 30 kya stimulated lineage diversification and greater interactions within and beyond Northern Sahul, to Australia, Wallacea, and beyond. These lineage dispersals did not, however, erase the strong geographic structuring of the maternal lineages visible in Northern Sahul, which persists to the present.

## Supplementary information

Figure S1

Figure S1

Figure S2

Figure S2

Figure S3

Figure S3

Figure S4

Figure S4

Table S1

Table S1

Table S2

Table S3

Table S4

Table S5
