# Supplementary figures and images for: Papuan mitochondrial genomes and the settlement of Sahul
Source: J Hum Genet. 2020 Jun 1;65(10):875–87. doi: 10.1038/s10038-020-0781-3 (PMC7449881; doi:10.1038/s10038-020-0781-3)

Figure S1. Maximum Parsimony Phylogenetic Tree of 607 Mitogenomes.

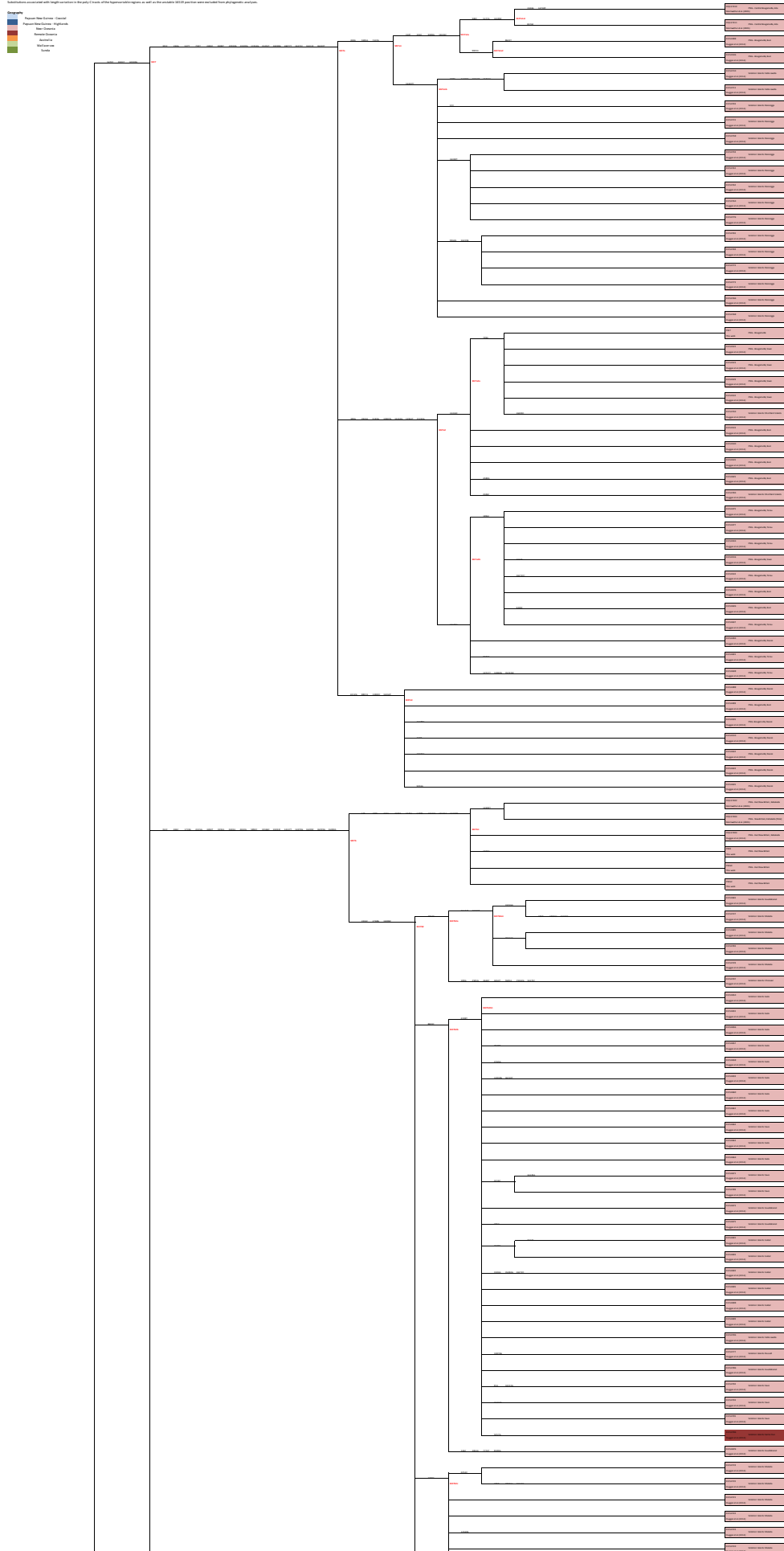

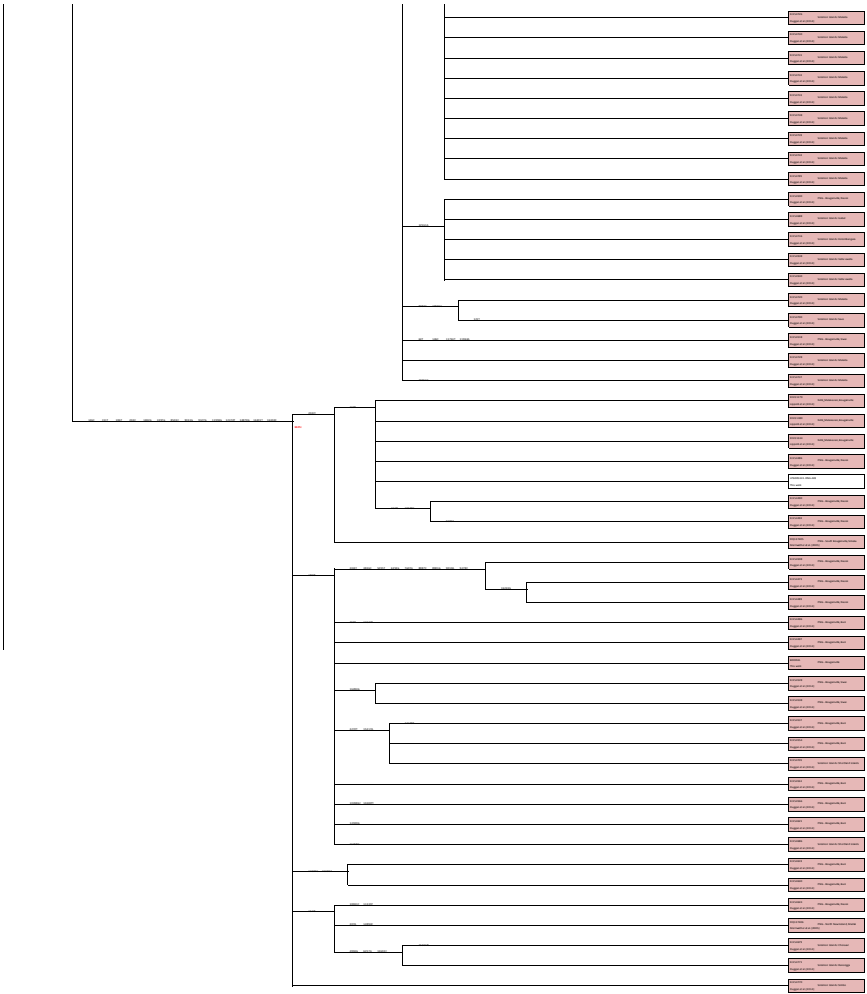

Supplement: Supplementary file 1 — Figure S1 [file 10038_2020_781_MOESM1_ESM.pdf]
