# Supplementary material for: Papuan mitochondrial genomes and the settlement of Sahul
Source: J Hum Genet. 2020 Jun 1;65(10):875–87. doi: 10.1038/s10038-020-0781-3 (PMC7449881; doi:10.1038/s10038-020-0781-3)

Figure S2. Maximum-Parsimony Phylogenetic Tree of M28 Mitogenomes.

Substitutions associated with length variation in the poly-C tracts of the hypervariable regions as well as the unstable 16519 position were excluded from phylogenetic analyses.

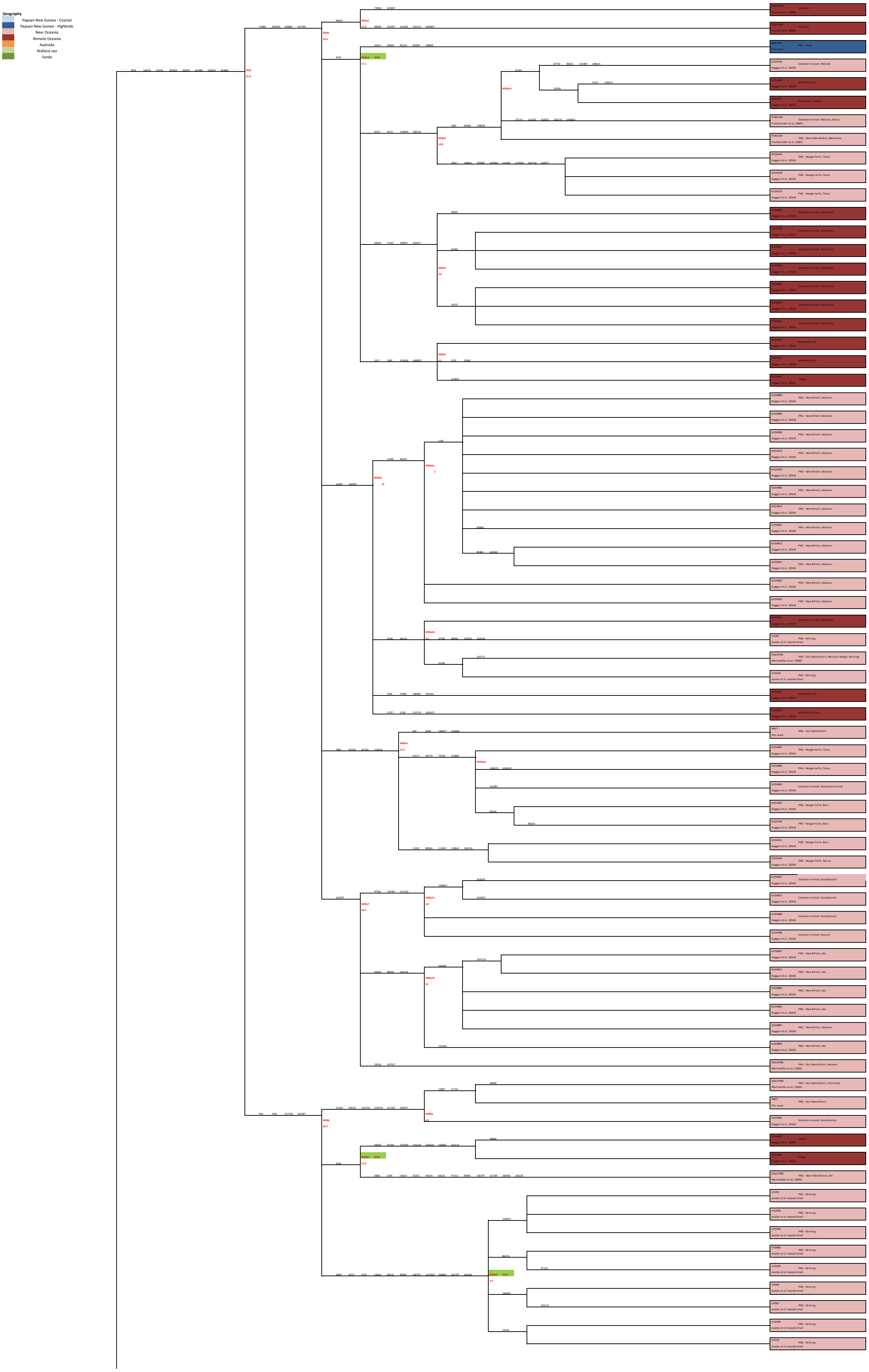

Supplement: Supplementary file 3 — Figure S2 [file 10038_2020_781_MOESM3_ESM.pdf]
