# Supplementary material for: Papuan mitochondrial genomes and the settlement of Sahul
Source: J Hum Genet. 2020 Jun 1;65(10):875–87. doi: 10.1038/s10038-020-0781-3 (PMC7449881; doi:10.1038/s10038-020-0781-3)

Figure S3. Maximum-Parsimony Phylogenetic Tree of M29'Q Mitogenomes.

Substitutions associated with length variation in the poly-C tracts of the hypervariable regions as well as the unstable 16519 position were excluded from phylogenetic analyses.

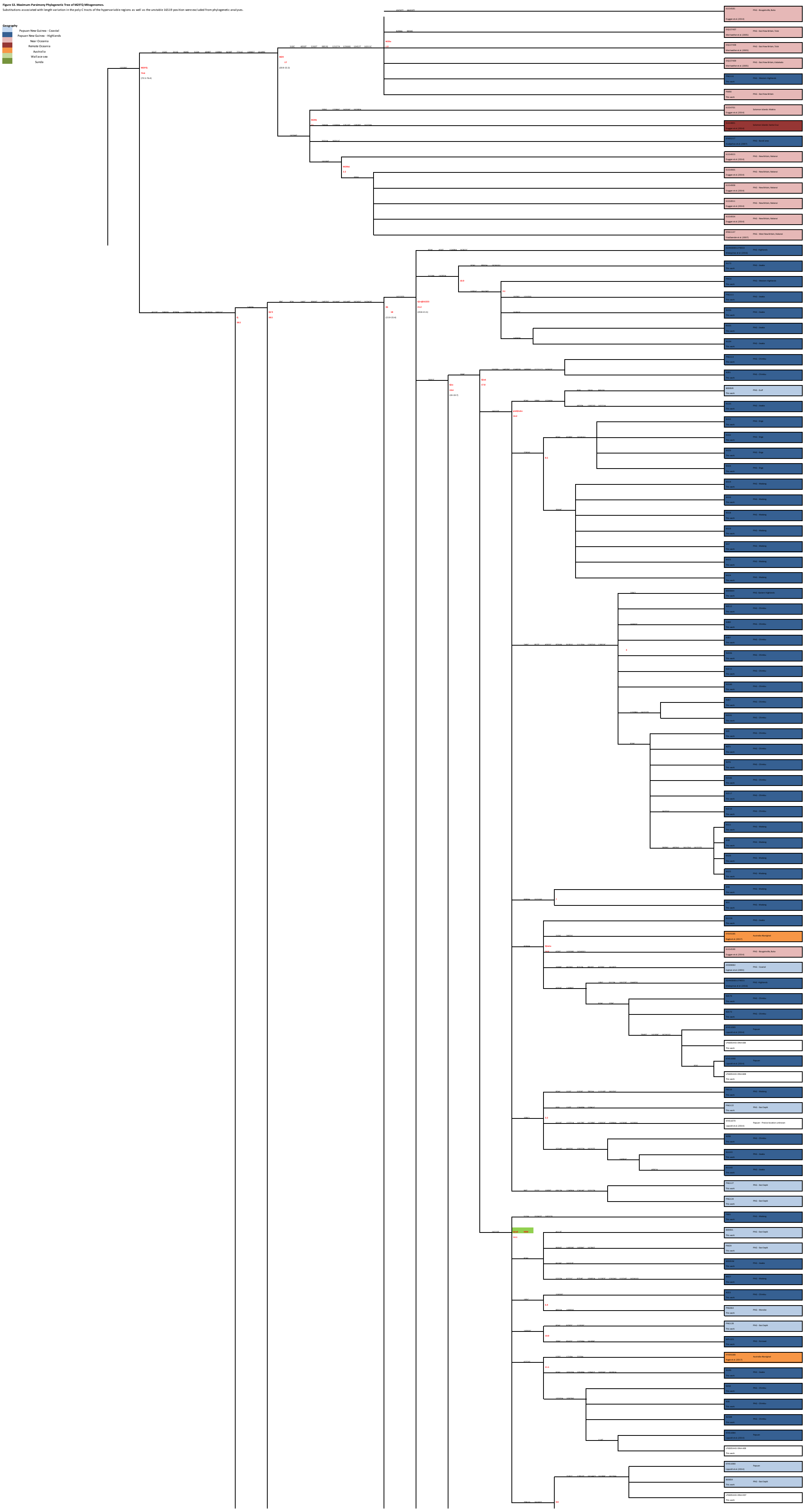

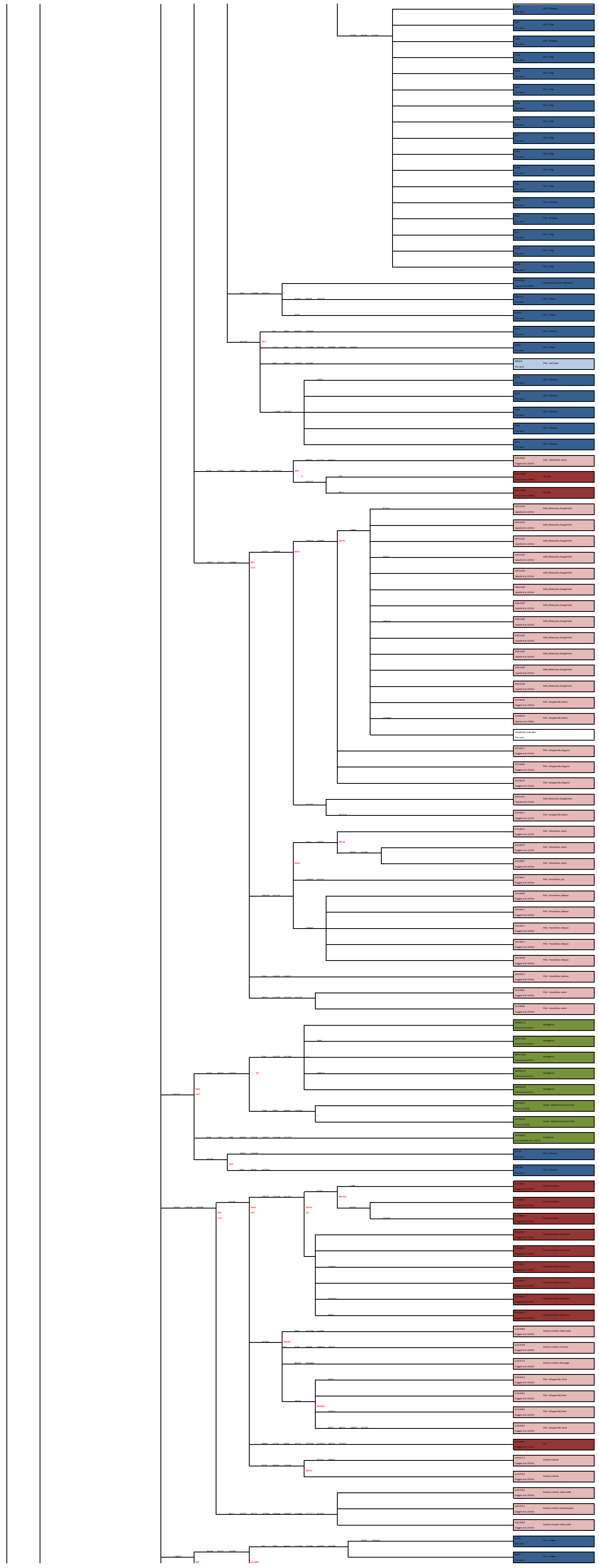

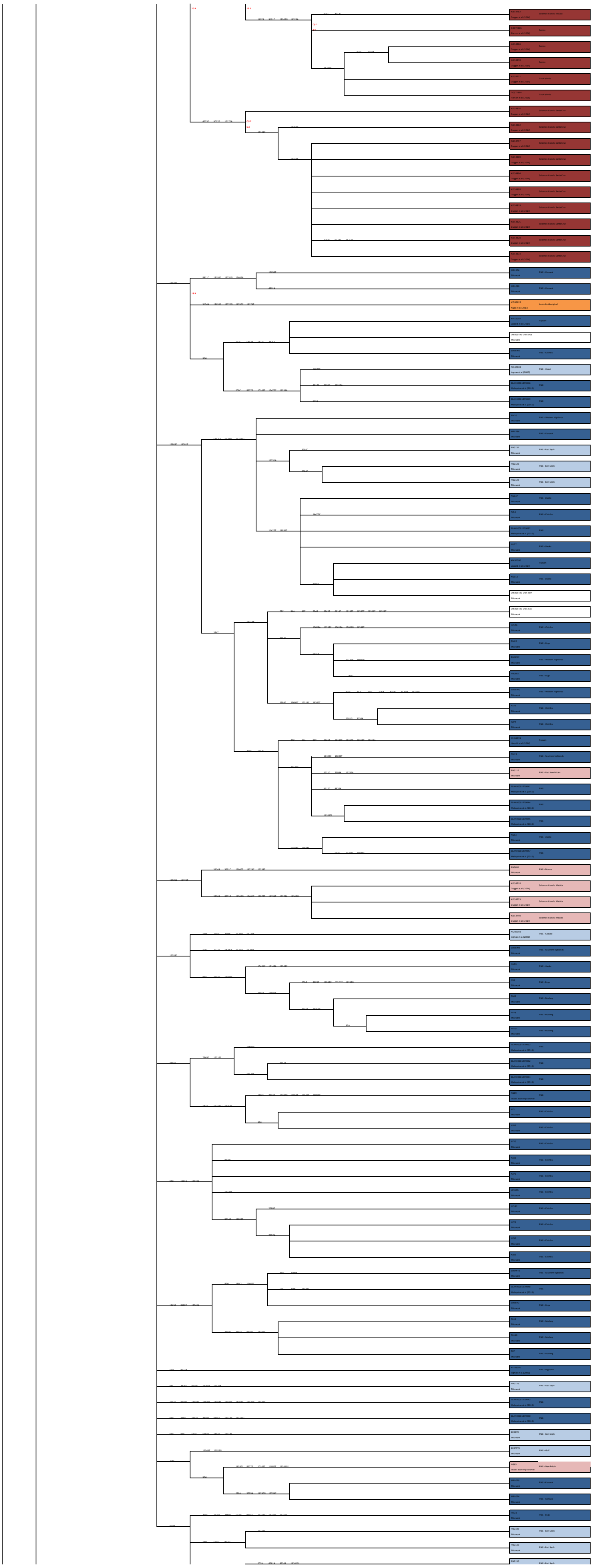
KJ154782
Solomon Islands: Tikopia
Duggan et al. (2014)
DQ372885
Samoa
Pierson et al. (2006)
KJ154206
Samoa
Duggan et al. (2014)
KJ154370
Samoa
Duggan et al. (2014)
KJ154211
Cook Islands
Duggan et al. (2014)
DQ372884
Cook Islands
Pierson et al. (2006)
KJ154816
Solomon Islands: Santa Cruz
Duggan et al. (2014)
KJ154802
Solomon Islands: Santa Cruz
Duggan et al. (2014)
KJ154797
Solomon Islands: Santa Cruz
Duggan et al. (2014)
KJ154800
Solomon Islands: Santa Cruz
Duggan et al. (2014)
KJ154804
Solomon Islands: Santa Cruz
Duggan et al. (2014)
KJ154808
Solomon Islands: Santa Cruz
Duggan et al. (2014)
KJ154819
Solomon Islands: Santa Cruz
Duggan et al. (2014)

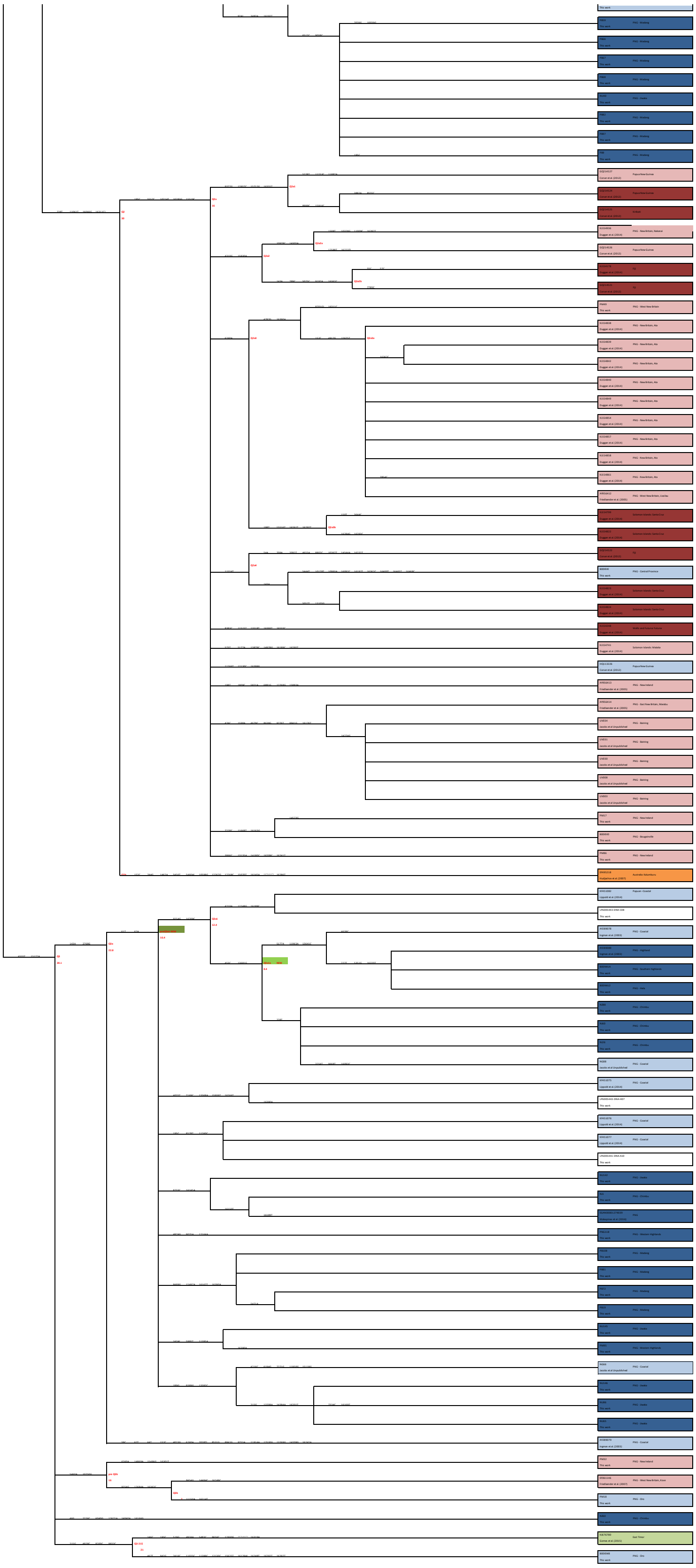

Supplement: Supplementary file 5 — Figure S3 [file 10038_2020_781_MOESM5_ESM.pdf]
