# Supplementary material for: Papuan mitochondrial genomes and the settlement of Sahul
Source: J Hum Genet. 2020 Jun 1;65(10):875–87. doi: 10.1038/s10038-020-0781-3 (PMC7449881; doi:10.1038/s10038-020-0781-3)
Supplement: Supplementary file 7 — Figure S4 [file 10038_2020_781_MOESM7_ESM.pdf]

Figure S4. Maximum-Parsimony Phylogenetic Tree of P Mitogenomes.

Substitutions associated with length variation in the poly-C tracts of the hypervariable regions as well as the unstable 16519 position were excluded from phylogenetic analyses.

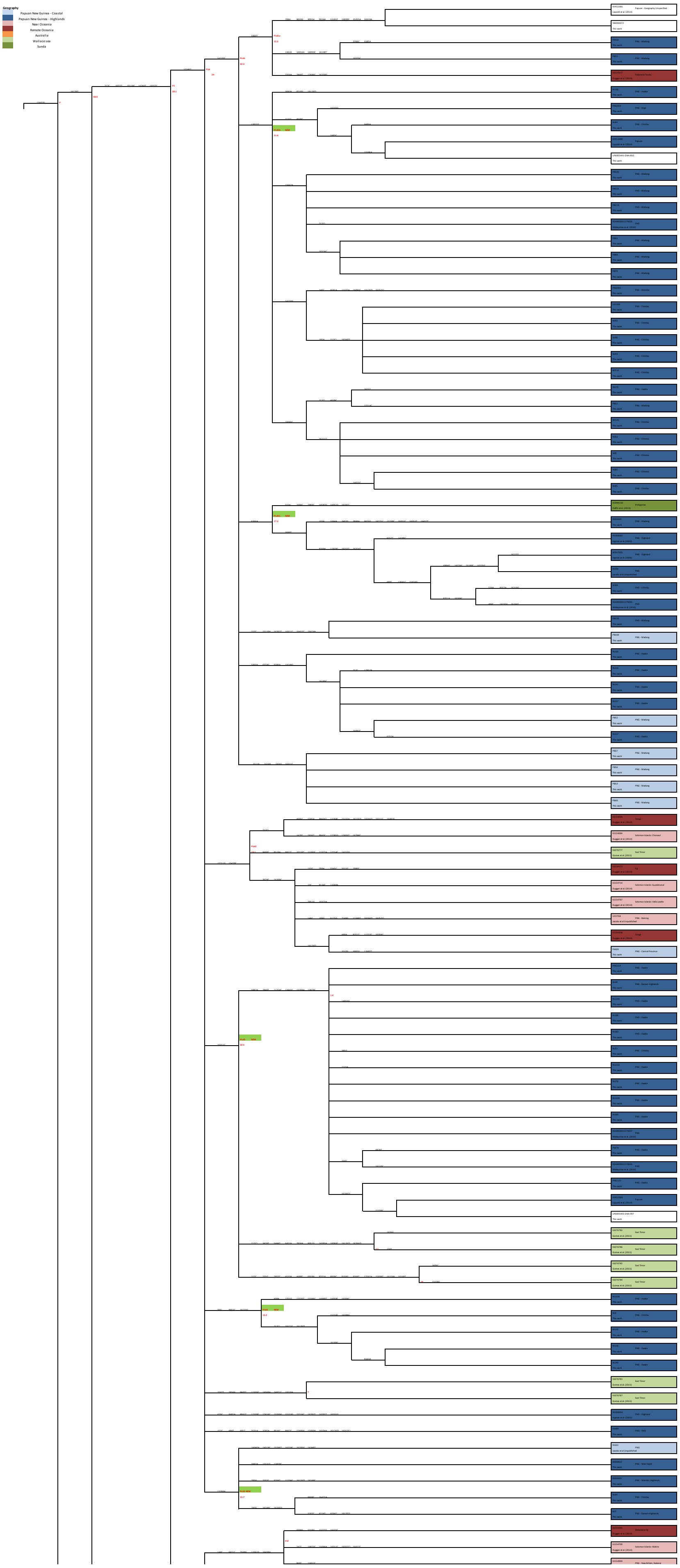

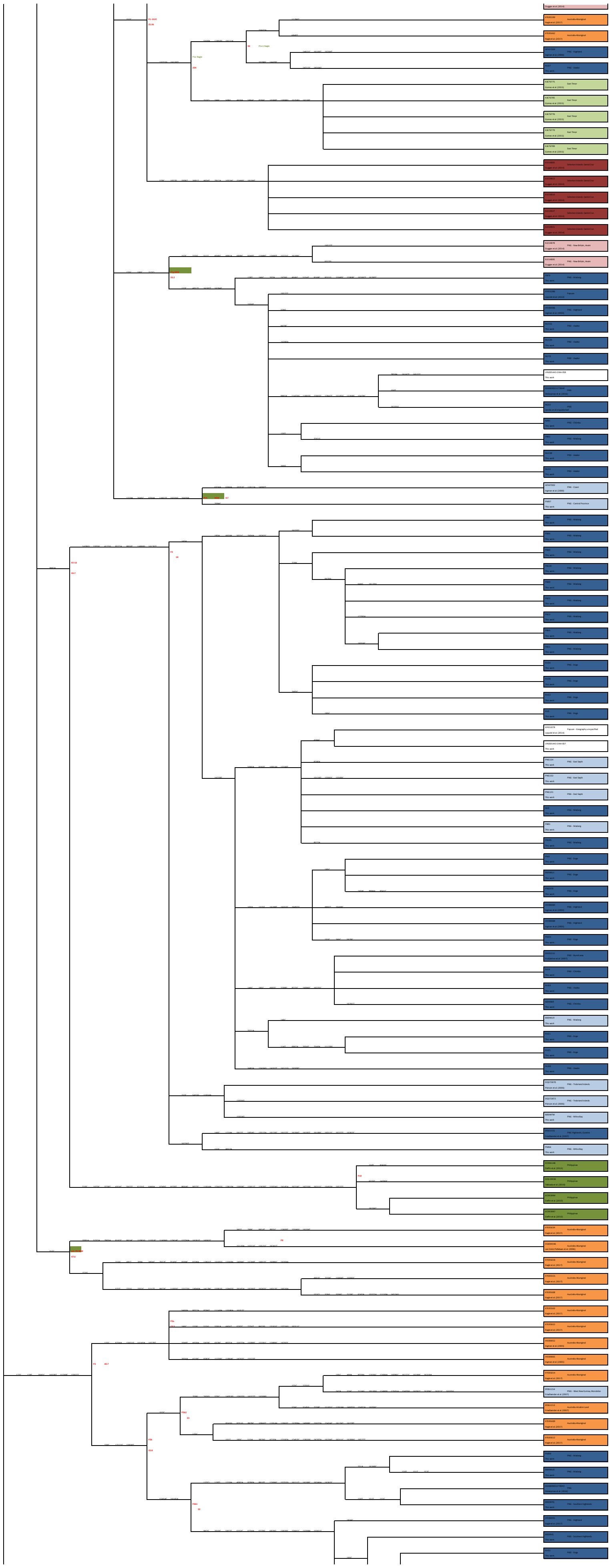

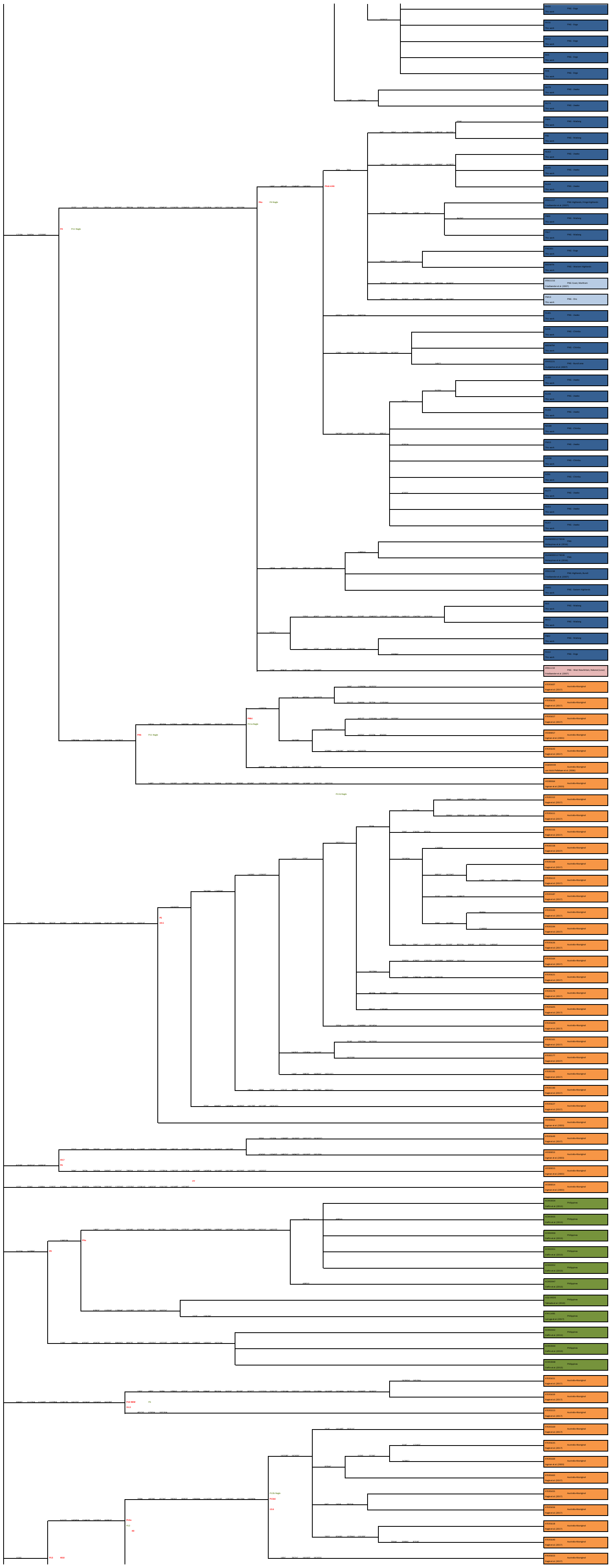

PM45 PNG - Eastern Highlands This work
MJ3 PNG - Madang This work
MJ12 PNG - Madang This work
PB02 PNG - Madang This work
NV14 PNG - Enga This work
EF061159 PNG - West New Britain, Nakanai (Loso) Friedlaender et al. (2007)
KY595607 Australia-Aboriginal Nagle et al. (2017)
KY595633 Australia-Aboriginal Nagle et al. (2017)
KY595657 Australia-Aboriginal Nagle et al. (2017)
AY289057 Australia-Aboriginal Ingman et al. (2003)
KY595643 Australia-Aboriginal Nagle et al. (2017)
DQ404444 Australia-Aboriginal van Holst Pellekaan et al. (2006)
AY289064 Australia-Aboriginal Ingman et al. (2003)
KY595552 Australia-Aboriginal Nagle et al. (2017)
KY595611 Australia-Aboriginal Nagle et al. (2017)
KY595556 Australia-Aboriginal Nagle et al. (2017)
KY595558 Australia-Aboriginal Nagle et al. (2017)
KY595568 Australia-Aboriginal Nagle et al. (2017)

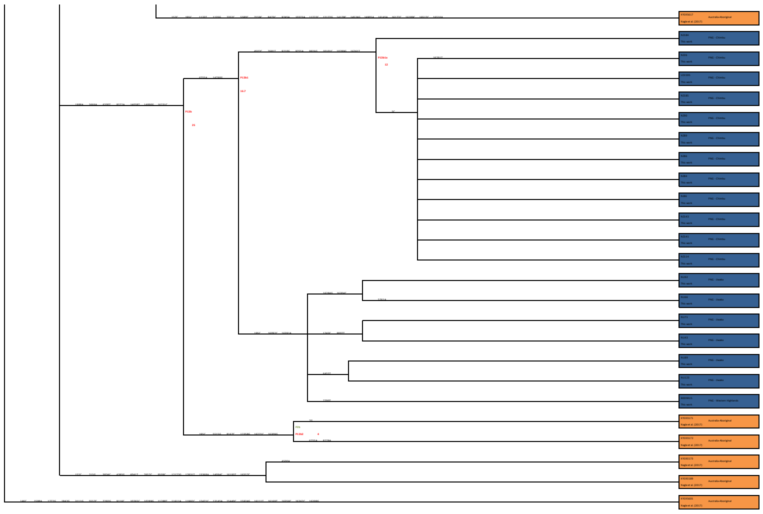

KY595617 Australia-Aboriginal Nagle et al. (2017)
N2184 PNG - Chimbu This work
N226 PNG - Chimbu This work
LO1595 PNG - Chimbu This work
N2185 PNG - Chimbu This work
N290 PNG - Chimbu This work
N289 PNG - Chimbu This work
N286 PNG - Chimbu This work
N284 PNG - Chimbu This work
NJ86 PNG - Chimbu This work
N2143 PNG - Chimbu This work
N2141 PNG - Chimbu This work
N2114 PNG - Chimbu This work
NU92 PNG - Jiwaka This work
NU66 PNG - Jiwaka This work
NU71 PNG - Jiwaka This work
NU43 PNG - Jiwaka This work
NU89 PNG - Jiwaka This work
NU123 PNG - Jiwaka This work
800IWU5 PNG - Western Highlands This work
KY595571 Australia-Aboriginal Nagle et al. (2017)
KY595572 Australia-Aboriginal Nagle et al. (2017)
KY595573 Australia-Aboriginal Nagle et al. (2017)
KY595589 Australia-Aboriginal Nagle et al. (2017)
KY595601 Australia-Aboriginal Nagle et al. (2017)
