# Supplementary material for: Papuan mitochondrial genomes and the settlement of Sahul
Source: J Hum Genet. 2020 Jun 1;65(10):875–87. doi: 10.1038/s10038-020-0781-3 (PMC7449881; doi:10.1038/s10038-020-0781-3)
Supplement: Supplementary file 9 — Table S1 [file 10038_2020_781_MOESM9_ESM.pdf]

Table S1. List of 379 new Papua New Guinean complete mitogenome sequences considered in this Study.

ID	Country	Province	District*	Village/region **	Population group / region	Source	Data generation ***	Haplogroup	Mutations relative to Rcrs
PB52	Papua New Guinea	Madang	Bogia	Bi Mat	Coastal/Lowland	This study - IMR collection	1	P1d1	A73G T212C A263G T310C A750G A1438G A2706G CN3106d A4769G G5460A C6077T A6359G C7028T A8860G G9380A T10118C G11719A C12346T A14148G C14766T A15326G A15607G
B00IEVD-PNG37	Papua New Guinea	Bougainville	Buka	ND	Near Oceania	This study - new collection	2	Q2a	A73G T195C G228T A263G T489C A750G A1438G A2706G T4117C A4769G G5460A T5557C A5843G C7028T T7220C A8701G G8790A A8860G T9540C C10214T A10283G A10398G C10400T T10873C C11061T C11668T G11719A C12705T G12940A T13500C C14766T T14783C G15043A G15301A A15326G T15519C A16066G G16129A A16163G C16223T A16241G T16519C
PM65	Papua New Guinea	Central Province	Abau	Gavnone	Coastal/Lowland	This study - new collection	1	B4a1a1	A73G T146C A263G CCCCCCT304d CA514d A750G A1438G A2706G A4769G T5465C T6719C C7028T A8860G G9123A T10238C G11719A C12239T A14022G C14766T A15326G A15746G
PM97	Papua New Guinea	Central Province	Kairuku	ND	Coastal/Lowland	This study - new collection	1	P1	A73G T212C A263G A750G A1438G G1719A A2706G A4769G C6077T C7028T T7094C T7645C G8764A A8860G T10118C G11719A A13927T C14766T A15326G A15607G C16176T A16235G
PM39	Papua New Guinea	Central Province	Goilala	Iluavi	Coastal/Lowland	This study - new collection	1	P1d2a	A73G T212C A263G A750G A1438G A2706G A3537G A3987G A4769G C6077T C7028T T7624C A8860G T10118C G11719A C12346T C12687T A12711G C14766T A15326G T15479C [illegible]
PM79	Papua New Guinea	Jiwaka	Banz	Koldor	Highlands	This study - new collection	1	P1d	A73G C150T T212C A263G T310d A750G A1438G A2706G A4769G C5987A C6077T C6878T C7028T C7849T A8860G T10118C T11254C G11719A C12346T T13443C G13708A T13879C [illegible]
B00IEW9-PNG101	Papua New Guinea	Chimbu	Kerowagi	ND	Highlands	This study - new collection	2	P2	A73G G143A T146C T195C A263G A750G A2706G A3203G G3882A A4122G A4769G T4907C C7028T A7298G T8222C G8572A C8859T A8860G G11719A C14766T A14890G A15326G
NJ85	Papua New Guinea	Chimbu	East Koroni	Kunabau	Highlands	This study - IMR collection	1	P1d1	A73G T212C A263G A750G A1438G A2706G A4769G C6077T C7028T A8860G T10118C G11719A C12346T C13071T T14025C C14766T A15326G A15607G T16093C C16111T C16176T [illegible]
PNG121	Papua New Guinea	East Sepik	Angoram	Ambolwari	Coastal/Lowland	This study - new collection	1	P2	A73G G143A A263G A750G A2706G A3203G G3882A A4122G A4769G G5046A C7028T G8572A C8859T A8860G C9293T G10914A G11719A C13749T C14766T A14890G A15326G [illegible]
PNG132	Papua New Guinea	East Sepik	Angoram	Kundiman	Coastal/Lowland	This study - new collection	1	P2	A73G G143A A263G A750G A2706G A3203G G3882A A4122G A4769G G5046A C7028T G8572A C8859T A8860G C9293T G10914A G11719A C13129T T13392C T13500C C13749T C14766T [illegible]
PNG134	Papua New Guinea	East Sepik	Angoram	Kundiman	Coastal/Lowland	This study - new collection	1	P2	A73G G143A A263G A750G A2706G A3203G G3882A A4122G A4769G G5046A C7028T G8290A G8572A C8859T A8860G C9293T G10914A G11719A C13749T C14766T A14890G A15326G
B00IEVQ-PNG88	Papua New Guinea	Southern highlands	Nipa-Kutubu	Porom	Highlands	This study - new collection	2	P3b	A73G T127C C128T T195C T236C A263G A750G A942G A1438G T2010C A2706G T3645C A4769G C5972T T6253C G6734A A6770G A6929G C7028T A8860G G11719A T12121C T13020C [illegible]
PNG001	Papua New Guinea	Enga	Kandep	ND	Highlands	This study - new collection	1	P4a1	G35A G36A A73G T146C T152C A263G A395G T507C A517G A750G A1438G G1719A A2706G G3915A T4216C A4769G C4814T G5460A T6407C C7028T G7852A A8860G A9587G G9755A [illegible]
PNG005	Papua New Guinea	Morobe	Menyamya	ND	Highlands	This study - new collection	1	P1d1	[illegible]
PNG031	Papua New Guinea	Manus	Manus	Pak islands	Near Oceania	This study - new collection	1	Q1	[illegible]
PNG057	Papua New Guinea	Enga	Wabag	Tinam	Highlands	This study - new collection	1	Q1	[illegible]
PNG059	Papua New Guinea	Enga	Wapenamanda	ND	Highlands	This study - new collection	1	P1d1	[illegible]
PNG076	Papua New Guinea	Enga	Kompiam	Konkan	Highlands	This study - new collection	1	P2	[illegible]
PM2	Papua New Guinea	Enga	Kompiam	Ambum	Highlands	This study - new collection	1	P2	[illegible]
PM25	Papua New Guinea	Enga	Kompiam	Ambum	Highlands	This study - new collection	1	P2	[illegible]
B00IWU2-PNG93	Papua New Guinea	Hela	ND	Yambaraka	Highlands	This study - new collection	2	Q3a1	[illegible]
PM89	Papua New Guinea	Hela	Komo-Magarima	ND	Highlands	This study - new collection	1	P1d	[illegible]
PM95	Papua New Guinea	Western Highlands	Tambul-Nebilyer	ND	Highlands	This study - new collection	1	Q3a+61 62	[illegible]
PNG114	Papua New Guinea	Jiwaka	Minz	Nokanum	Highlands	This study - new collection	1	P1d	[illegible]
PNG115	Papua New Guinea	Jiwaka	Gumine	Kumai	Highlands	This study - new collection	1	P1d	[illegible]
B00IWU8-PNG80	Papua New Guinea	Madang	Madang	ND	Highlands	This study - new collection	2	P3b	[illegible]
PM80	Papua New Guinea	Madang	ND	ND	Coastal/Lowland	This study - new collection	1	P3b	[illegible]
PB02	Papua New Guinea	Madang	Middle Ramu	Ainonk	Highlands	This study - IMR collection	1	P4a	[illegible]
PB108	Papua New Guinea	Madang	Middle Ramu	Wowo	Highlands	This study - IMR collection	1	Q3a+61 62	[illegible]
PB11	Papua New Guinea	Madang	Middle Ramu	Sanguyak	Highlands	This study - IMR collection	1	P1d1	[illegible]
PB110	Papua New Guinea	Madang	Middle Ramu	Wowo	Highlands	This study - IMR collection	1	Q1	[illegible]
PB119	Papua New Guinea	Madang	Middle Ramu	Wowo	Highlands	This study - IMR collection	1	P1d1	[illegible]
PB125	Papua New Guinea	Madang	Middle Ramu	Bi Lum	Highlands	This study - IMR collection	1	P1d1	[illegible]
PB13	Papua New Guinea	Madang	Middle Ramu	Sanguyak	Highlands	This study - IMR collection	1	P2	[illegible]
PB15	Papua New Guinea	Madang	Middle Ramu	Sanguyak	Highlands	This study - IMR collection	1	Q1	[illegible]
PB17	Papua New Guinea	Madang	Middle Ramu	Sanguyak	Highlands	This study - IMR collection	1	P4a1	[illegible]
PB23	Papua New Guinea	Madang	Middle Ramu	Salemp	Highlands	This study - IMR collection	1	P2	[illegible]
PB5	Papua New Guinea	Madang	Middle Ramu	Sanguyak	Highlands	This study - IMR collection	1	P4a1	[illegible]
PB64	Papua New Guinea	Madang	Middle Ramu	Salemp	Highlands	This study - IMR collection	1	P2	[illegible]
PB66	Papua New Guinea	Madang	Middle Ramu	Salemp	Highlands	This study - IMR collection	1	Q1	[illegible]
PB7	Papua New Guinea	Madang	Middle Ramu	ND	Highlands	This study - IMR collection	1	Q1	[illegible]
PB77	Papua New Guinea	Madang	Middle Ramu	Salemp	Highlands	This study - IMR collection	1	Q1a1	[illegible]
PB81	Papua New Guinea	Madang	Middle Ramu	Ainonk	Highlands	This study - IMR collection	1	Q3a+61 62	[illegible]
PB86	Papua New Guinea	Madang	Middle Ramu	Sanguyak	Highlands	This study - IMR collection	1	P2	[illegible]
PB88	Papua New Guinea	Madang	Middle Ramu	Ainonk	Highlands	This study - IMR collection	1	P1d1	[illegible]
PB89	Papua New Guinea	Madang	Middle Ramu	ND	Highlands	This study - IMR collection	1	P2	[illegible]
PB9	Papua New Guinea	Madang	Middle Ramu	Sanguyak	Highlands	This study - IMR collection	1	Q1	[illegible]
PB95	Papua New Guinea	Madang	Middle Ramu	Ainonk	Highlands	This study - IMR collection	1	Q1a1	[illegible]
PB99	Papua New Guinea	Madang	Middle Ramu	ND	Highlands	This study - IMR collection	1	P4a1	[illegible]
NZ1	Papua New Guinea	Chimbu	karimui-Nomane	Naio	Highlands	This study - IMR collection	1	Q1	[illegible]
NZ10	Papua New Guinea	Chimbu	karimui-Nomane	Di Ile	Highlands	This study - IMR collection	1	Q1a1	[illegible]
NZ112	Papua New Guinea	Chimbu	karimui-Nomane	Moio	Highlands	This study - IMR collection	1	Q1a1	[illegible]
NZ114	Papua New Guinea	Chimbu	karimui-Nomane	Naio	Highlands	This study - IMR collection	1	P	[illegible]
[illegible]	[illegible]	[illegible]	[illegible]	[illegible]	[illegible]	[illegible]	[illegible]	[illegible]	[illegible]
NZ141	Papua New Guinea	Chimbu	karimui-Nomane	Waio	Highlands	This study - IMR collection	1	P	[illegible]
NZ142	Papua New Guinea	Chimbu	karimui-Nomane	Waio	Highlands	This study - IMR collection	1	P1d1	[illegible]
NZ143	Papua New Guinea	Chimbu	karimui-Nomane	Waio	Highlands	This study - IMR collection	1	P	[illegible]
NZ170	Papua New Guinea	Chimbu	karimui-Nomane	Yuro	Highlands	This study - IMR collection	1	Q1	[illegible]
NZ173	Papua New Guinea	Chimbu	karimui-Nomane	Naio	Highlands	This study - IMR collection	1	Q1a1a	[illegible]
NZ176	Papua New Guinea	Chimbu	karimui-Nomane	Kiribari	Highlands	This study - IMR collection	1	Q1d	[illegible]
NZ179	Papua New Guinea	Chimbu	karimui-Nomane	Kiribari	Highlands	This study - IMR collection	1	Q1a1a	[illegible]
NZ180	Papua New Guinea	Chimbu	karimui-Nomane	Boi Samalu	Highlands	This study - IMR collection	1	Q1a1	[illegible]
NZ182	Papua New Guinea	Chimbu	karimui-Nomane	Kiribari	Highlands	This study - IMR collection	1	Q1	[illegible]
NZ184	Papua New Guinea	Chimbu	karimui-Nomane	Masi	Highlands	This study - IMR collection	1	P	[illegible]
NZ185	Papua New Guinea	Chimbu	karimui-Nomane	Kiribari	Highlands	This study - IMR collection	1	P	[illegible]
NZ186	Papua New Guinea	Chimbu	karimui-Nomane	Boi Samalu	Highlands	This study - IMR collection	1	Q1a1	[illegible]
NZ188	Papua New Guinea	Chimbu	karimui-Nomane	ND	Highlands	This study - IMR collection	1	Q1a1	[illegible]
NZ189	Papua New Guinea	Chimbu	karimui-Nomane	ND	Highlands	This study - IMR collection	1	P4a1	[illegible]
NZ191	Papua New Guinea	Chimbu	karimui-Nomane	ND	Highlands	This study - IMR collection	1	Q1a1	[illegible]
NZ20	Papua New Guinea	Chimbu	karimui-Nomane	Haia	Highlands	This study - IMR collection	1	Q1	[illegible]
NZ206	Papua New Guinea	Chimbu	karimui-Nomane	Yaweho	Highlands	This study - IMR collection	1	Q1a1	[illegible]
NZ208	Papua New Guinea	Chimbu	karimui-Nomane	Hwaiyo	Highlands	This study - IMR collection	1	P4a1	[illegible]
NZ21	Papua New Guinea	Chimbu	karimui-Nomane	Moio	Highlands	This study - IMR collection	1	Q1	[illegible]
NZ210	Papua New Guinea	Chimbu	karimui-Nomane	Naio	Highlands	This study - IMR collection	1	Q1a1	[illegible]
NZ211	Papua New Guinea	Chimbu	karimui-Nomane	Di Ile	Highlands	This study - IMR collection	1	Q1a1	[illegible]
NZ217	Papua New Guinea	Chimbu	karimui-Nomane	ND	Highlands	This study - IMR collection	1	Q1a1	[illegible]
NZ23	Papua New Guinea	Chimbu	karimui-Nomane	Hwaiyo	Highlands	This study - IMR collection	1	Q1a1	[illegible]
NZ26	Papua New Guinea	Chimbu	karimui-Nomane	Di Ile	Highlands	This study - IMR collection	1	P	[illegible]
NZ28	Papua New Guinea	Chimbu	karimui-Nomane	Yaweho	Highlands	This study - IMR collection	1	P4a1	[illegible]
NZ3	Papua New Guinea	Chimbu	karimui-Nomane	Naio	Highlands	This study - IMR collection	1	Q1a1	[illegible]
NZ32	Papua New Guinea	Chimbu	karimui-Nomane	Di Ile	Highlands	This study - IMR collection	1	Q1	[illegible]
NZ42	Papua New Guinea	Chimbu	karimui-Nomane	Naio	Highlands	This study - IMR collection	1	Q1	[illegible]
[illegible]	[illegible]	[illegible]	[illegible]	[illegible]	[illegible]	[illegible]	[illegible]	[illegible]	[illegible]
NZ6	Papua New Guinea	Chimbu	karimui-Nomane	Hwaiyo	Highlands	This study - IMR collection	1	Q1a1	[illegible]
NZ60	Papua New Guinea	Chimbu	karimui-Nomane	ND	Highlands	This study - IMR collection	1	Q3	[illegible]
NZ62	Papua New Guinea	Chimbu	karimui-Nomane	Hwaiyo	Highlands	This study - IMR collection	1	P1d1	[illegible]
NZ67	Papua New Guinea	Chimbu	karimui-Nomane	Naio	Highlands	This study - IMR collection	1	Q1a1	[illegible]
NZ70	Papua New Guinea	Chimbu	karimui-Nomane	Moio	Highlands	This study - IMR collection	1	Q1	[illegible]
NZ71	Papua New Guinea	Chimbu	karimui-Nomane	Moio	Highlands	This study - IMR collection	1	Q1a1	[illegible]
NZ72	Papua New Guinea	Chimbu	karimui-Nomane	Di Ile	Highlands	This study - IMR collection	1	Q1	[illegible]
NZ76	Papua New Guinea	Chimbu	karimui-Nomane	Moio	Highlands	This study - IMR collection	1	Q1a1	[illegible]
NZ77	Papua New Guinea	Chimbu	karimui-Nomane	Di Ile	Highlands	This study - IMR collection	1	Q1	[illegible]
NZ80	Papua New Guinea	Chimbu	karimui-Nomane	Waio	Highlands	This study - IMR collection	1	Q1a1	[illegible]
NZ81	Papua New Guinea	Chimbu	karimui-Nomane	Yaweho	Highlands	This study - IMR collection	1	P1	[illegible]
NZ82	Papua New Guinea	Chimbu	karimui-Nomane	Hwaiyo	Highlands	This study - IMR collection	1	Q1	[illegible]
NZ84	Papua New Guinea	Chimbu	karimui-Nomane	Moio	Highlands	This study - IMR collection	1	P	[illegible]
NZ85	Papua New Guinea	Chimbu	karimui-Nomane	Moio	Highlands	This study - IMR collection	1	P1d1	[illegible]
NZ86	Papua New Guinea	Chimbu	karimui-Nomane	Naio	Highlands	This study - IMR collection	1	P	[illegible]
NZ87	Papua New Guinea	Chimbu	karimui-Nomane	Hwaiyo	Highlands	This study - IMR collection	1	Q1a1	[illegible]
NZ88	Papua New Guinea	Chimbu	karimui-Nomane	Yaweho	Highlands	This study - IMR collection	1	Q3a1	[illegible]
NZ89	Papua New Guinea	Chimbu	karimui-Nomane	Moio	Highlands	This study - IMR collection	1	P	[illegible]
NZ9	Papua New Guinea	Chimbu	karimui-Nomane	Di Ile	Highlands	This study - IMR collection	1	P1d1	[illegible]
NZ90	Papua New Guinea	Chimbu	karimui-Nomane	Naio	Highlands	This study - IMR collection	1	P	[illegible]
NZ91	Papua New Guinea	Chimbu	karimui-Nomane	Naio	Highlands	This study - IMR collection	1	Q1a1	[illegible]
NZ92	Papua New Guinea	Chimbu	karimui-Nomane	Naio	Highlands	This study - IMR collection	1	P1d1	[illegible]
NZ96	Papua New Guinea	Chimbu	karimui-Nomane	Hwaiyo	Highlands	This study - IMR collection	1	P4a1	[illegible]
NZ97	Papua New Guinea	Chimbu	karimui-Nomane	Yaweho	Highlands	This study - IMR collection	1	Q1	[illegible]
NZ98	Papua New Guinea	Chimbu	karimui-Nomane	Hwaiyo	Highlands	This study - IMR collection	1	P1d1	[illegible]
NZ99	Papua New Guinea	Chimbu	karimui-Nomane	Naio	Highlands	This study - IMR collection	1	Q1a1a	[illegible]
NJ1	Papua New Guinea	Chimbu	karimui-Nomane	Yuro	Highlands	This study - IMR collection	1	Q3a+61 62	[illegible]
NJ22	Papua New Guinea	Chimbu	karimui-Nomane	Yuro	Highlands	This study - IMR collection	1	P1d	[illegible]
NJ24	Papua New Guinea	Chimbu	karimui-Nomane	Yuro	Highlands	This study - IMR collection	1	P2	[illegible]
NJ26	Papua New Guinea	Chimbu	karimui-Nomane	Yuro	Highlands	This study - IMR collection	1	Q3a1	[illegible]
NJ3	Papua New Guinea	Chimbu	karimui-Nomane	Yuro	Highlands	This study - IMR collection	1	P1d	[illegible]
NJ44	Papua New Guinea	Chimbu	karimui-Nomane	Karimui Station	Highlands	This study - IMR collection	1	P1d1	[illegible]
NJ52	Papua New Guinea	Chimbu	karimui-Nomane	Naio	Highlands	This study - IMR collection	1	Q1a	[illegible]
NJ53	Papua New Guinea	Chimbu	karimui-Nomane	Waio	Highlands	This study - IMR collection	1	Q1	[illegible]
NJ60	Papua New Guinea	Chimbu	karimui-Nomane	Boi Samalu	Highlands	This study - IMR collection	1	Q3a1	[illegible]
NJ86	Papua New Guinea	Chimbu	karimui-Nomane	Naio	Highlands	This study - IMR collection	1	P	[illegible]
NJ90	Papua New Guinea	Chimbu	karimui-Nomane	Waio	Highlands	This study - IMR collection	1	P1d1	[illegible]
NJ95	Papua New Guinea	Chimbu	karimui-Nomane	Karimui Station	Highlands	This study - IMR collection	1	P1d	[illegible]
NJ48	Papua New Guinea	Eastern Highlands	ND	Loa, Ari Ki Yuh	Highlands	This study - IMR collection	1	P1d	[illegible]
PB49	Papua New Guinea	Madang	Bogia	Goibang	Coastal/Lowland	This study - IMR collection	1	P1d1	[illegible]
PB53	Papua New Guinea	Madang	Bogia	Goibang	Coastal/Lowland	This study - IMR collection	1	P1d1	[illegible]
PB56	Papua New Guinea	Madang	Bogia	Goibang	Coastal/Lowland	This study - IMR collection	1	P1d1	[illegible]
PB57	Papua New Guinea	Madang	Bogia	Goibang	Coastal/Lowland	This study - IMR collection	1	P1d1	[illegible]
B00IEW0-PNG34	Papua New Guinea	Madang	Madang	ND	Coastal/Lowland	This study - new collection	2	P1d1	[illegible]
PM58	Papua New Guinea	Madang	Middle Ramu	Simbai	Highlands	This study - new collection	1	P1d1a	[illegible]
PNG003	Papua New Guinea	Manus	Manus	Los Negros Rural	Near Oceania	This study - new collection	1	B4a1a1	[illegible]
PM66	Papua New Guinea	Milne Bay	Kiriwini-Goodenough	ND	Coastal/Lowland	This study - new collection	1	P2	[illegible]
NU37	Papua New Guinea	Jiwaka	Jimi	Tsi Ngoropa	Highlands	This study - IMR collection	1	P1d1	[illegible]
NU38	Papua New Guinea	Jiwaka	Jimi	Tsi Ngoropa	Highlands	This study - IMR collection	1	P1d1	[illegible]
NU41	Papua New Guinea	Jiwaka	Jimi	Tsi Ngoropa	Highlands	This study - IMR collection	1	P1d1	[illegible]
NU54	Papua New Guinea	Jiwaka	Jimi	Karap	Highlands	This study - IMR collection	1	P1d1	[illegible]
NU57	Papua New Guinea	Jiwaka	Jimi	Maegmol	Highlands	This study - IMR collection	1	P1d1	[illegible]
NU64	Papua New Guinea	Jiwaka	Jimi	Tsi Ngoropa	Highlands	This study - IMR collection	1	P1d1	[illegible]
NU67	Papua New Guinea	Jiwaka	Jimi	Koriom	Highlands	This study - IMR collection	1	P1+152	[illegible]
NU75	Papua New Guinea	Jiwaka	Jimi	Ongomol	Highlands	This study - IMR collection	1	P1d1	[illegible]
PNG064	Papua New Guinea	Morobe	Tewae-Siassi	ND	Coastal/Lowland	This study - new collection	1	Q1a1	[illegible]
PM49	Papua New Guinea	West New Britain	Talasea	Kimbe	Near Oceania	This study - new collection	1	Q2a3	[illegible]
PM17	Papua New Guinea	New Ireland	Kavieng	Mangai	Near Oceania	This study - new collection	1	Q2a	[illegible]
PM32	Papua New Guinea	New Ireland	Kavieng	Tikana rural, Djaul island	Near Oceania	This study - new collection	1	Q3	[illegible]
PM86	Papua New Guinea	New Ireland	Kavieng	Lavongai Rural	Near Oceania	This study - new collection	1	Q2a	[illegible]
NJ7	Papua New Guinea	Eastern Highlands	Okapa	North Fore	Highlands	This study - IMR collection	1	P1d	[illegible]
B00IEW8-PNG24	Papua New Guinea	Oro	Popondetta	Maisin	Coastal/Lowland	This study - new collection	2	Q3	[illegible]
[illegible]	[illegible]	[illegible]	[illegible]	[illegible]	[illegible]	[illegible]	[illegible]	[illegible]	[illegible]
PM18	Papua New Guinea	Oro	Sohe	Gona	Coastal/Lowland	This study - new collection	1	Q3b	[illegible]
L01596	Papua New Guinea	Chimbu	Karimui-Nomane	Pio Tura	Highlands	This study - IMR collection	1	P1d1	[illegible]

LO1586	Papua New Guinea	Chimbu	Karimui-Nomane	Pio Tura	Highlands	This study - IMR collection	1	Q1	[illegible]
LO1595	Papua New Guinea	Chimbu	Karimui-Nomane	Pio Tura	Highlands	This study - IMR collection	1	P	[illegible]
PB104	Papua New Guinea	Madang	Madang	Saker-Ganus, Yoidik	Coastal/Lowland	This study - IMR collection	1	P1d1	[illegible]
B00IWTQ-PNG19	Papua New Guinea	Southern highlands	Ialibu-Pangia	ND	Highlands	This study - new collection	2	P3b	[illegible]
B00IWUA-PNG35	Papua New Guinea	Southern highlands	Ialibu-Pangia	Maia	Highlands	This study - new collection	2	Q3a1	[illegible]
PB85	Papua New Guinea	Madang	Bogia	Urawin, Angaba	Coastal/Lowland	This study - IMR collection	1	P2	[illegible]
MJ1	Papua New Guinea	Madang	Middle Ramu	Yilu (W Schraders)	Highlands	This study - IMR collection	1	Q1a1a	[illegible]
MJ11	Papua New Guinea	Madang	Middle Ramu	Yilu (W Schraders)	Highlands	This study - IMR collection	1	Q1a1	[illegible]
MJ12	Papua New Guinea	Madang	Middle Ramu	Yilu (W Schraders)	Highlands	This study - IMR collection	1	P4a	[illegible]
MJ14	Papua New Guinea	Madang	Middle Ramu	Yilu (W Schraders)	Highlands	This study - IMR collection	1	Q1a1	[illegible]
MJ16	Papua New Guinea	Madang	Middle Ramu	Yilu (W Schraders)	Highlands	This study - IMR collection	1	Q1a1	[illegible]
MJ17	Papua New Guinea	Madang	Middle Ramu	Yilu (W Schraders)	Highlands	This study - IMR collection	1	Q1a1	[illegible]
MJ18	Papua New Guinea	Madang	Middle Ramu	Yilu (W Schraders)	Highlands	This study - IMR collection	1	Q1a1	[illegible]
MJ19	Papua New Guinea	Madang	Middle Ramu	Yilu (W Schraders)	Highlands	This study - IMR collection	1	Q1a1	[illegible]
MJ20	Papua New Guinea	Madang	Middle Ramu	Yilu (W Schraders)	Highlands	This study - IMR collection	1	Q1a1	[illegible]
MJ22	Papua New Guinea	Madang	Middle Ramu	Yilu (W Schraders)	Highlands	This study - IMR collection	1	Q1a1	[illegible]
MJ23	Papua New Guinea	Madang	Middle Ramu	Yilu (W Schraders)	Highlands	This study - IMR collection	1	Q1a1	[illegible]
MJ24	Papua New Guinea	Madang	Middle Ramu	Yilu (W Schraders)	Highlands	This study - IMR collection	1	Q1a1	[illegible]
MJ3	Papua New Guinea	Madang	Middle Ramu	Yilu (W Schraders)	Highlands	This study - IMR collection	1	P4a	[illegible]
MJ6	Papua New Guinea	Madang	Middle Ramu	Yilu (W Schraders)	Highlands	This study - IMR collection	1	Q1a1a	[illegible]
MJ7	Papua New Guinea	Madang	Middle Ramu	Yilu (W Schraders)	Highlands	This study - IMR collection	1	Q1a1	[illegible]
MJ8	Papua New Guinea	Madang	Middle Ramu	Yilu (W Schraders)	Highlands	This study - IMR collection	1	Q1a1	[illegible]
MJ9	Papua New Guinea	Madang	Middle Ramu	Yilu (W Schraders)	Highlands	This study - IMR collection	1	P2	[illegible]
PB101	Papua New Guinea	Madang	Middle Ramu	Sangapi	Highlands	This study - IMR collection	1	Q1	[illegible]
PB105	Papua New Guinea	Madang	Middle Ramu	Wulim	Highlands	This study - IMR collection	1	P2	[illegible]
PB120	Papua New Guinea	Madang	Middle Ramu	Aradimp	Highlands	This study - IMR collection	1	P2	[illegible]
PB121	Papua New Guinea	Madang	Middle Ramu	Aradimp	Highlands	This study - IMR collection	1	P1d1	[illegible]
PB136	Papua New Guinea	Madang	Middle Ramu	Wuli	Highlands	This study - IMR collection	1	P1d1	[illegible]
PB139	Papua New Guinea	Madang	Middle Ramu	Sangapi	Highlands	This study - IMR collection	1	Q1a1a	[illegible]
PB19	Papua New Guinea	Madang	Middle Ramu	Sangapi	Highlands	This study - IMR collection	1	Q3a+61 62	[illegible]
PB21	Papua New Guinea	Madang	Middle Ramu	Gebrau	Highlands	This study - IMR collection	1	P2	[illegible]
PB25	Papua New Guinea	Madang	Middle Ramu	Dusin	Highlands	This study - IMR collection	1	Q1a1	[illegible]
PB47	Papua New Guinea	Madang	Middle Ramu	Dusin	Highlands	This study - IMR collection	1	Q1a1	[illegible]
PB48	Papua New Guinea	Madang	Middle Ramu	Gebrau	Highlands	This study - IMR collection	1	Q1a	[illegible]
PB55	Papua New Guinea	Madang	Middle Ramu	Gebrau	Highlands	This study - IMR collection	1	P1d1a	[illegible]
PB59	Papua New Guinea	Madang	Middle Ramu	Sangapi	Highlands	This study - IMR collection	1	Q1	[illegible]
PB65	Papua New Guinea	Madang	Middle Ramu	Gebrau	Highlands	This study - IMR collection	1	Q1	[illegible]
PB67	Papua New Guinea	Madang	Middle Ramu	Gebrau	Highlands	This study - IMR collection	1	Q1	[illegible]
PB68	Papua New Guinea	Madang	Middle Ramu	Gebrau	Highlands	This study - IMR collection	1	Q1	[illegible]
PB69	Papua New Guinea	Madang	Middle Ramu	Wulim	Highlands	This study - IMR collection	1	Q1a	[illegible]
PB70	Papua New Guinea	Madang	Middle Ramu	Gebrau	Highlands	This study - IMR collection	1	Q1a	[illegible]
PB71	Papua New Guinea	Madang	Middle Ramu	Gebrau	Highlands	This study - IMR collection	1	Q1a	[illegible]
PB72	Papua New Guinea	Madang	Middle Ramu	Fankfank	Highlands	This study - IMR collection	1	Q3a+61 62	[illegible]
PB73	Papua New Guinea	Madang	Middle Ramu	Gebrau	Highlands	This study - IMR collection	1	Q1a	[illegible]
PB74	Papua New Guinea	Madang	Middle Ramu	Dusin	Highlands	This study - IMR collection	1	P1d1	[illegible]
PB80	Papua New Guinea	Madang	Middle Ramu	Gebrau	Highlands	This study - IMR collection	1	P2	[illegible]
PB82	Papua New Guinea	Madang	Middle Ramu	Gebrau	Highlands	This study - IMR collection	1	Q1	[illegible]
PB83	Papua New Guinea	Madang	Middle Ramu	Dusin	Highlands	This study - IMR collection	1	Q1a1	[illegible]
PB87	Papua New Guinea	Madang	Middle Ramu	Sangapi	Highlands	This study - IMR collection	1	P2	[illegible]
PB91	Papua New Guinea	Madang	Middle Ramu	Dusin	Highlands	This study - IMR collection	1	P1d1	[illegible]
PB92	Papua New Guinea	Madang	Middle Ramu	Gebrau	Highlands	This study - IMR collection	1	P1	[illegible]
PB94	Papua New Guinea	Madang	Middle Ramu	Gebrau	Highlands	This study - IMR collection	1	P4a1	[illegible]
PB97	Papua New Guinea	Madang	Middle Ramu	Sangapi	Highlands	This study - IMR collection	1	Q1	[illegible]
PB76	Papua New Guinea	Madang	Middle Ramu	Gebrau	Highlands	This study - IMR collection	1	P1	[illegible]
PB78	Papua New Guinea	Madang	Middle Ramu	Gebrau	Highlands	This study - IMR collection	1	Q1	[illegible]
NV1	Papua New Guinea	Enga	Kompiam	Wapi, Liti Ankama	Highlands	This study - IMR collection	1	Q1a1	[illegible]
NV10	Papua New Guinea	Enga	Kompiam	Wapi, Liti Ankama	Highlands	This study - IMR collection	1	Q1a1	[illegible]
NV11	Papua New Guinea	Enga	Kompiam	Wapi, Liti Ankama	Highlands	This study - IMR collection	1	Q1a1	[illegible]
NV12	Papua New Guinea	Enga	Kompiam	Wapi, Liti Ankama	Highlands	This study - IMR collection	1	P3b	[illegible]
NV13	Papua New Guinea	Enga	Kompiam	Wapi, Liti Ankama	Highlands	This study - IMR collection	1	P2	[illegible]
NV14	Papua New Guinea	Enga	Kompiam	Wapi, Liti Ankama	Highlands	This study - IMR collection	1	P4a	[illegible]
NV16	Papua New Guinea	Enga	Kompiam	Wapi, Liti Ankama	Highlands	This study - IMR collection	1	P3b	[illegible]
NV18	Papua New Guinea	Enga	Kompiam	Wapi, Liti Ankama	Highlands	This study - IMR collection	1	Q1a1	[illegible]
NV19	Papua New Guinea	Enga	Kompiam	Wapi, Liti Ankama	Highlands	This study - IMR collection	1	Q1a1	[illegible]
NV2	Papua New Guinea	Enga	Kompiam	Wapi, Liti Ankama	Highlands	This study - IMR collection	1	Q1a1	[illegible]
NV20	Papua New Guinea	Enga	Kompiam	Wapi, Liti Ankama	Highlands	This study - IMR collection	1	P3b	[illegible]
NV21	Papua New Guinea	Enga	Kompiam	Wapi, Liti Ankama	Highlands	This study - IMR collection	1	P2	[illegible]
NV23	Papua New Guinea	Enga	Kompiam	Wapi, Liti Ankama	Highlands	This study - IMR collection	1	Q1a1	[illegible]
NV24	Papua New Guinea	Enga	Kompiam	Wapi, Liti Ankama	Highlands	This study - IMR collection	1	Q1a1a	[illegible]
NV25	Papua New Guinea	Enga	Kompiam	Wapi, Liti Ankama	Highlands	This study - IMR collection	1	P2	[illegible]
NV26	Papua New Guinea	Enga	Kompiam	Wapi, Liti Ankama	Highlands	This study - IMR collection	1	Q1a1	[illegible]
NV27	Papua New Guinea	Enga	Kompiam	Wapi, Liti Ankama	Highlands	This study - IMR collection	1	Q1a1	[illegible]
NV28	Papua New Guinea	Enga	Kompiam	Wapi, Liti Ankama	Highlands	This study - IMR collection	1	P2	[illegible]
NV29	Papua New Guinea	Enga	Kompiam	Wapi, Liti Ankama	Highlands	This study - IMR collection	1	Q1a1a	[illegible]
NV3	Papua New Guinea	Enga	Kompiam	Wapi, Liti Ankama	Highlands	This study - IMR collection	1	P2	[illegible]
NV30	Papua New Guinea	Enga	Kompiam	Wapi, Liti Ankama	Highlands	This study - IMR collection	1	Q1a1	[illegible]
NV31	Papua New Guinea	Enga	Kompiam	Wapi, Liti Ankama	Highlands	This study - IMR collection	1	P3b	[illegible]
NV32	Papua New Guinea	Enga	Kompiam	Wapi, Liti Ankama	Highlands	This study - IMR collection	1	Q1a1a	[illegible]
NV33	Papua New Guinea	Enga	Kompiam	Wapi, Liti Ankama	Highlands	This study - IMR collection	1	Q1a1a	[illegible]
NV34	Papua New Guinea	Enga	Kompiam	Wapi, Liti Ankama	Highlands	This study - IMR collection	1	P2	[illegible]
NV36	Papua New Guinea	Enga	Kompiam	Wapi, Liti Ankama	Highlands	This study - IMR collection	1	Q1a1	[illegible]
NV4	Papua New Guinea	Enga	Kompiam	Wapi, Liti Ankama	Highlands	This study - IMR collection	1	Q1	[illegible]
NV5	Papua New Guinea	Enga	Kompiam	Wapi, Liti Ankama	Highlands	This study - IMR collection	1	P3b	[illegible]
NV6	Papua New Guinea	Enga	Kompiam	Wapi, Liti Ankama	Highlands	This study - IMR collection	1	P3b	[illegible]
NV7	Papua New Guinea	Enga	Kompiam	Wapi, Liti Ankama	Highlands	This study - IMR collection	1	Q1a1	[illegible]
NV9	Papua New Guinea	Enga	Kompiam	Wapi, Liti Ankama	Highlands	This study - IMR collection	1	Q1a1	[illegible]
B00IEWV-PNG46	Papua New Guinea	Western Highlands	Mount Hagen	Molga	Highlands	This study - new collection	2	P1d	[illegible]
NU142	Papua New Guinea	Jiwaka	Jimi	Kol, upper Jimi	Highlands	This study - IMR collection	1	Q3a+61 62	[illegible]
NU145	Papua New Guinea	Jiwaka	Jimi	Kol, upper Jimi	Highlands	This study - IMR collection	1	Q3a+61 62	[illegible]
PNG118	Papua New Guinea	Western Highlands	Mount Hagen	ND	Highlands	This study - new collection	1	Q3a+61 62	[illegible]
B00IWU3-PNG94	Papua New Guinea	West Sepik	Telefomin	Oksapmin	Highlands	This study - new collection	2	P1d	[illegible]
B00IEV8-PNG84	Papua New Guinea	Bougainville	South Bougainville	Siwai	Near Oceania	This study - new collection	2	B4a1a1a1c	[illegible]
B00IEV9-PNG55	Papua New Guinea	Bougainville	South Bougainville	Siwai	Near Oceania	This study - new collection	2	B4a1a1a	[illegible]
B00IEVA-PNG83	Papua New Guinea	Bougainville	South Bougainville	ND	Near Oceania	This study - new collection	2	M27c	[illegible]
B00IEVB-PNG98	Papua New Guinea	Bougainville	South Bougainville	ND	Near Oceania	This study - new collection	2	B4a1a1a	[illegible]
B00IEVE-PNG54	Papua New Guinea	Central Province	Abau	Mailu Island	Coastal/Lowland	This study - new collection	2	Q2a4	[illegible]
B00IEVF-PNG48	Papua New Guinea	Central Province	Abau	Aroma coast	Coastal/Lowland	This study - new collection	2	B4a1a1a	[illegible]
B00IEVG-PNG61	Papua New Guinea	Central Province	Kairuku-Hiri	ND	Coastal/Lowland	This study - new collection	2	B4a1a1	[illegible]
B00IEVH-PNG70	Papua New Guinea	Central Province	Rigo	Hula	Coastal/Lowland	This study - new collection	2	B4a1a1	[illegible]
B00IEVI-PNG52	Papua New Guinea	East Sepik	Ambuti	Waskua	Coastal/Lowland	This study - new collection	2	Q1a1	[illegible]
B00IEVJ-PNG99	Papua New Guinea	East Sepik	Wewak	Haniak	Coastal/Lowland	This study - new collection	2	Q1a	[illegible]
B00IEVK-PNG43	Papua New Guinea	East Sepik	Ambuti	ND	Coastal/Lowland	This study - new collection	2	Q1	[illegible]
B00IEVL-PNG21	Papua New Guinea	East Sepik	Wewak	ND	Coastal/Lowland	This study - new collection	2	Q1a1	[illegible]
B00IEVM-PNG100	Papua New Guinea	Eastern Highlands	Kainantu	Kainantu	Highlands	This study - new collection	2	R14	[illegible]
B00IEVN-PNG36	Papua New Guinea	Central Province	ND	Kivovi	Coastal/Lowland	This study - new collection	2	B4a1a1a	[illegible]
B00IEVO-PNG82	Papua New Guinea	Enga	Kompiam	Yampu	Highlands	This study - new collection	2	Q1	[illegible]
B00IEVR-PNG69	Papua New Guinea	Gulf	Kikori	Orokolo	Coastal/Lowland	This study - new collection	2	Q1a1a	[illegible]
B00IEVW-PNG12	Papua New Guinea	Jiwaka	ND	ND	Highlands	This study - new collection	2	Q1a1	[illegible]
B00IEW1-PNG71	Papua New Guinea	Milne Bay	Alotau	Suau	Coastal/Lowland	This study - new collection	2	E1a1b4	[illegible]
B00IEW3-PNG30	Papua New Guinea	Milne Bay	Esa'ala	Normanby island	Coastal/Lowland	This study - new collection	2	B4a1a1	[illegible]
B00IEW5-PNG68	Papua New Guinea	Milne Bay	Alotau	Wamira	Coastal/Lowland	This study - new collection	2	B4a1a1	[illegible]
B00IEWA-PNG81	Papua New Guinea	Chimbu	Sina Sina-Yonggomugl	Dinga	highlands	This study - new collection	2	Q1	[illegible]
B00IEWD-PNG67	Papua New Guinea	Southern highlands	Kagua-Erave	ND	highlands	This study - new collection	2	Q1	[illegible]
B00IEWG-PNG4	Papua New Guinea	Western Highlands	Mount Hagen	Kuli	highlands	This study - new collection	2	Q1	[illegible]
B00IEWH-PNG50	Papua New Guinea	Eastern Highlands	Unggai-Benna	Kerenaga	Highlands	This study - new collection	2	Q1a	[illegible]
B00IEWJ-PNG11	Papua New Guinea	Western Highlands	Mount Hagen	Mount Hagen	Highlands	This study - new collection	2	Q1	[illegible]
B00IEWM-PNG91	Papua New Guinea	Bougainville	Buin	Kugaiogu	Coastal/Lowland	This study - new collection	2	B4a1a1	[illegible]
B00IEWP-PNG38	Papua New Guinea	Central Province	Kairuku-Hiri	Pinu	Coastal/Lowland	This study - new collection	2	E1a2+(16261)	[illegible]
B00IEWR-PNG51	Papua New Guinea	Enga	Lagaip-Porgera	ND	Highlands	This study - new collection	2	M28a+204	[illegible]
B00IWTM-PNG10A	Papua New Guinea	Chimbu	Gumine	Moromaule	Highlands	This study - new collection	2	P4a1	[illegible]
B00IWTN-PNG14	Papua New Guinea	Western Highlands	Mount Hagen	Mount Hagen	Highlands	This study - new collection	2	P4a1	[illegible]
B00IWTR-PNG22	Papua New Guinea	Gulf	Kerema	Mewari	Coastal/Lowland	This study - new collection	2	Q1	[illegible]
B00IWTT-PNG26	Papua New Guinea	Milne Bay	Alotau	Dreta	Coastal/Lowland	This study - new collection	2	B4a1a1+16126	[illegible]
B00IWTU-PNG33	Papua New Guinea	Southern highlands	Nipa-Kutubu	Poroma	Highlands	This study - new collection	2	Q1	[illegible]
[illegible]	[illegible]	[illegible]	[illegible]	[illegible]	[illegible]	[illegible]	[illegible]	[illegible]	[illegible]
B00IWTX-PNG63	Papua New Guinea	Madang	Sumkar	Karkar island	Near Oceania	This study - new collection	2	B4a1a1a	[illegible]
B00IWU1-PNG87	Papua New Guinea	Enga	Wabag	Rakamanda	Highlands	This study - new collection	2	P2	[illegible]
B00IWU5-PNG102	Papua New Guinea	Western Highlands	Mount Hagen	Mount Hagen	Highlands	This study - new collection	2	P	[illegible]
B00IWU7-PNG9	Papua New Guinea	West Sepik	ND	ND	Coastal/Lowland	This study - new collection	2	E1a2a1	[illegible]
PM103	Papua New Guinea	Morobe	ND	ND	Coastal/Lowland	This study - new collection	1	B4a1a1+151	[illegible]
PM104	Papua New Guinea	Oro	Popondetta	ND	Coastal/Lowland	This study - new collection	1	B4a1a1	[illegible]
PM13	Papua New Guinea	Oro	Popondetta	ND	Coastal/Lowland	This study - new collection	1	P4a1	[illegible]
PM15	Papua New Guinea	Jiwaka	ND	ND	Highlands	This study - new collection	1	P4a1	[illegible]
PM16	Papua New Guinea	Western Highlands	Mount Hagen	Mount Hagen	Highlands	This study - new collection	1	Q1+@16223	[illegible]
PM20	Papua New Guinea	Western Highlands	Mount Hagen	Mount Hagen	Highlands	This study - new collection	1	Q1	[illegible]
PM23	Papua New Guinea	Enga	Wabag	ND	Highlands	This study - new collection	1	Q1	[illegible]
PM27	Papua New Guinea	East New Britain	Kokopo	Tingnanalom	Near Oceania	This study - new collection	1	M28b1	[illegible]
PM28	Papua New Guinea	Oro	Popondetta	ND	Coastal/Lowland	This study - new collection	1	B4a1a1a	[illegible]
PM29	Papua New Guinea	East Sepik	Wewak	Kereau islands	Coastal/Lowland	This study - new collection	1	Q1a1	[illegible]

PM40	Papua New Guinea	East New Britain	Kokopo	Raluana	Near Oceania	This study - new collection	1	B4a1a1a	[illegible]
PM45	Papua New Guinea	Eastern Highlands	Unggai-Benna	Wantrifa	Highlands	This study - new collection	1	P4a	[illegible]
PM56	Papua New Guinea	New Ireland	Kavieng	Kabil	Near Oceania	This study - new collection	1	B4a1a1	[illegible]
PM6	Papua New Guinea	Bougainville	Bana	Lagovis	Near Oceania	This study - new collection	1	B4a1a1x	[illegible]
PM60	Papua New Guinea	East New Britain	Kokopo	Ravat	Near Oceania	This study - new collection	1	M27b1	[illegible]
PM62	Papua New Guinea	East New Britain	Kokopo	Karavi	Near Oceania	This study - new collection	1	M27b1	[illegible]
PM7	Papua New Guinea	Bougainville	Siwai	Haisi	Near Oceania	This study - new collection	1	M27a2a	[illegible]
PM72	Papua New Guinea	Milne Bay	Kiriwini-Goodenough	Okheboma	Coastal/Lowland	This study - new collection	1	B4a1a1	[illegible]
PM73	Papua New Guinea	Milne Bay	Alotau	ND	Coastal/Lowland	This study - new collection	1	B4a1a1	[illegible]
PM74	Papua New Guinea	Southern highlands	Mendi-Munihu	Konjop	Highlands	This study - new collection	1	Q1	[illegible]
PM75	Papua New Guinea	East New Britain	Gazelle	ND	Near Oceania	This study - new collection	1	B4a1a1a19	[illegible]
PM77	Papua New Guinea	East New Britain	Gazelle	ND	Near Oceania	This study - new collection	1	M28a6	[illegible]
PM78	Papua New Guinea	New Ireland	Kavieng	ND	Near Oceania	This study - new collection	1	B4a1a1	[illegible]
PM8	Papua New Guinea	East New Britain	Kokopo	ND	Near Oceania	This study - new collection	1	M27b1	[illegible]
PM82	Papua New Guinea	Enga	Kompiam	Yampu	Highlands	This study - new collection	1	Q1	[illegible]
PM90	Papua New Guinea	East New Britain	Gazelle	ND	Near Oceania	This study - new collection	1	M29a	[illegible]
PM92	Papua New Guinea	West New Britain	Talasea	Kololo	Near Oceania	This study - new collection	1	B4a1a1a	[illegible]
NU104	Papua New Guinea	Jiwaka	Jimi	Kol, upper Jimi	Highlands	This study - IMR collection	1	P1d	[illegible]
NU113	Papua New Guinea	Jiwaka	Jimi	Kol, upper Jimi	Highlands	This study - IMR collection	1	Q1	[illegible]
NU123	Papua New Guinea	Jiwaka	Jimi	Kol, upper Jimi	Highlands	This study - IMR collection	1	P	[illegible]
NU124	Papua New Guinea	Jiwaka	Jimi	Kol, upper Jimi	Highlands	This study - IMR collection	1	P1d	[illegible]
NU133	Papua New Guinea	Jiwaka	Jimi	Kol, upper Jimi	Highlands	This study - IMR collection	1	P1d	[illegible]
NU134	Papua New Guinea	Jiwaka	Jimi	Kol, upper Jimi	Highlands	This study - IMR collection	1	M73a	[illegible]
NU136	Papua New Guinea	Jiwaka	Jimi	Kol, upper Jimi	Highlands	This study - IMR collection	1	Q1a1a	[illegible]
NU137	Papua New Guinea	Jiwaka	Jimi	Kol, upper Jimi	Highlands	This study - IMR collection	1	Q1	[illegible]
NU138	Papua New Guinea	Jiwaka	Jimi	Kol, upper Jimi	Highlands	This study - IMR collection	1	P1	[illegible]
NU139	Papua New Guinea	Jiwaka	Jimi	Kol, upper Jimi	Highlands	This study - IMR collection	1	P1	[illegible]
NU140	Papua New Guinea	Jiwaka	Jimi	Kol, upper Jimi	Highlands	This study - IMR collection	1	Q1a1a	[illegible]
NU143	Papua New Guinea	Jiwaka	Jimi	Kol, upper Jimi	Highlands	This study - IMR collection	1	Q1a1a	[illegible]
NU146	Papua New Guinea	Jiwaka	Jimi	Kol, upper Jimi	Highlands	This study - IMR collection	1	Q3a+61 62	[illegible]
NU149	Papua New Guinea	Jiwaka	Jimi	Kol, upper Jimi	Highlands	This study - IMR collection	1	P1d	[illegible]
NU151	Papua New Guinea	Jiwaka	Jimi	Kol, upper Jimi	Highlands	This study - IMR collection	1	Q1a	[illegible]
NU152	Papua New Guinea	Jiwaka	Jimi	Kol, upper Jimi	Highlands	This study - IMR collection	1	P1	[illegible]
NU27	Papua New Guinea	Jiwaka	Jimi	Kwiop, middle Jimi	Highlands	This study - IMR collection	1	Q1	[illegible]
NU34	Papua New Guinea	Jiwaka	Jimi	Tsi Ngoropa, middle Jimi	Highlands	This study - IMR collection	1	Q1+@16223	[illegible]
NU35	Papua New Guinea	Jiwaka	Jimi	Kwiop, middle Jimi	Highlands	This study - IMR collection	1	Q1a1a	[illegible]
NU36	Papua New Guinea	Jiwaka	Jimi	Kwiop, middle Jimi	Highlands	This study - IMR collection	1	Q1f	[illegible]
NU39	Papua New Guinea	Jiwaka	Jimi	Tabi Buga, middle Jimi	Highlands	This study - IMR collection	1	Q1	[illegible]
NU40	Papua New Guinea	Jiwaka	Jimi	Tsi Ngoropa, middle Jimi	Highlands	This study - IMR collection	1	Q1	[illegible]
NU42	Papua New Guinea	Jiwaka	Jimi	Tabi Buga, middle Jimi	Highlands	This study - IMR collection	1	P1	[illegible]
NU43	Papua New Guinea	Jiwaka	Jimi	Tsi Ngoropa, middle Jimi	Highlands	This study - IMR collection	1	P	[illegible]
NU44	Papua New Guinea	Jiwaka	Jimi	Kwiop, middle Jimi	Highlands	This study - IMR collection	1	P4a1	[illegible]
NU45	Papua New Guinea	Jiwaka	Jimi	Tsi Ngoropa, middle Jimi	Highlands	This study - IMR collection	1	P4a1	[illegible]
NU46	Papua New Guinea	Jiwaka	Jimi	Mogini, middle Jimi	Highlands	This study - IMR collection	1	Q1a1	[illegible]
NU47	Papua New Guinea	Jiwaka	Jimi	Tabi Buga, middle Jimi	Highlands	This study - IMR collection	1	P4a1	[illegible]
NU48	Papua New Guinea	Jiwaka	Jimi	Tsi Ngoropa, middle Jimi	Highlands	This study - IMR collection	1	P4a1	[illegible]
NU49	Papua New Guinea	Jiwaka	Jimi	Kwiop, middle Jimi	Highlands	This study - IMR collection	1	P1d	[illegible]
NU50	Papua New Guinea	Jiwaka	Jimi	Tabi Buga, middle Jimi	Highlands	This study - IMR collection	1	Q1a	[illegible]
NU51	Papua New Guinea	Jiwaka	Jimi	Tsi Ngoropa, middle Jimi	Highlands	This study - IMR collection	1	P4a1	[illegible]
NU53	Papua New Guinea	Jiwaka	Jimi	Tsi Ngoropa, middle Jimi	Highlands	This study - IMR collection	1	Q1	[illegible]
NU55	Papua New Guinea	Jiwaka	Jimi	Tabi Buga, middle Jimi	Highlands	This study - IMR collection	1	Q1+@16223	[illegible]
NU56	Papua New Guinea	Jiwaka	Jimi	Tabi Buga, middle Jimi	Highlands	This study - IMR collection	1	Q1+@16223	[illegible]
NU58	Papua New Guinea	Jiwaka	Jimi	Koriom, middle Jimi	Highlands	This study - IMR collection	1	P1d	[illegible]
NU59	Papua New Guinea	Jiwaka	Jimi	Korendiu, middle Jimi	Highlands	This study - IMR collection	1	P1d	[illegible]
NU60	Papua New Guinea	Jiwaka	Jimi	Kwiop, middle Jimi	Highlands	This study - IMR collection	1	P1d	[illegible]
NU62	Papua New Guinea	Jiwaka	Jimi	Tolli, middle Jimi	Highlands	This study - IMR collection	1	P4a1	[illegible]
NU63	Papua New Guinea	Jiwaka	Jimi	Amuliba, middle Jimi	Highlands	This study - IMR collection	1	P2	[illegible]
NU65	Papua New Guinea	Jiwaka	Jimi	Kwiop, middle Jimi	Highlands	This study - IMR collection	1	Q3a+61 62	[illegible]
NU66	Papua New Guinea	Jiwaka	Jimi	Korendiu, middle Jimi	Highlands	This study - IMR collection	1	P	[illegible]
NU68	Papua New Guinea	Jiwaka	Jimi	Korendiu, middle Jimi	Highlands	This study - IMR collection	1	P4a1	[illegible]
NU69	Papua New Guinea	Jiwaka	Jimi	Tabi Buga, middle Jimi	Highlands	This study - IMR collection	1	P2	[illegible]
NU70	Papua New Guinea	Jiwaka	Jimi	Ongomol, middle Jimi	Highlands	This study - IMR collection	1	P1	[illegible]
NU71	Papua New Guinea	Jiwaka	Jimi	Maegmol, middle Jimi	Highlands	This study - IMR collection	1	P	[illegible]
NU73	Papua New Guinea	Jiwaka	Jimi	Ongomol, middle Jimi	Highlands	This study - IMR collection	1	Q1+@16223	[illegible]
NU74	Papua New Guinea	Jiwaka	Jimi	Ongomol, middle Jimi	Highlands	This study - IMR collection	1	P3b	[illegible]
NU76	Papua New Guinea	Jiwaka	Jimi	Ongomol, middle Jimi	Highlands	This study - IMR collection	1	P1d	[illegible]
NU77	Papua New Guinea	Jiwaka	Jimi	Maegmol, middle Jimi	Highlands	This study - IMR collection	1	P4a1	[illegible]
NU79	Papua New Guinea	Jiwaka	Jimi	Karap, middle Jimi	Highlands	This study - IMR collection	1	P3b	[illegible]
NU80	Papua New Guinea	Jiwaka	Jimi	Ongomol, middle Jimi	Highlands	This study - IMR collection	1	P4a1	[illegible]
NU83	Papua New Guinea	Jiwaka	Jimi	Ongomol, middle Jimi	Highlands	This study - IMR collection	1	P1d	[illegible]
NU85	Papua New Guinea	Jiwaka	Jimi	Ongomol, middle Jimi	Highlands	This study - IMR collection	1	P4a1	[illegible]
NU86	Papua New Guinea	Jiwaka	Jimi	Ongomol, middle Jimi	Highlands	This study - IMR collection	1	Q3a+61 62	[illegible]
NU88	Papua New Guinea	Jiwaka	Jimi	Maegmol, middle Jimi	Highlands	This study - IMR collection	1	P1d	[illegible]
NU89	Papua New Guinea	Jiwaka	Jimi	Ongomol, middle Jimi	Highlands	This study - IMR collection	1	P	[illegible]
NU90	Papua New Guinea	Jiwaka	Jimi	Ongomol, middle Jimi	Highlands	This study - IMR collection	1	Q1f	[illegible]
NU92	Papua New Guinea	Jiwaka	Jimi	Ongomol, middle Jimi	Highlands	This study - IMR collection	1	P	[illegible]
PNG109	Papua New Guinea	East Sepik	Yangoru-Saussia	ND	Coastal/Lowland	This study - new collection	1	Q1	[illegible]
PNG111	Papua New Guinea	Jiwaka	Anglimp-South Waghi	ND	Highlands	This study - new collection	1	Q1+@16223	[illegible]
PNG112	Papua New Guinea	Jiwaka	Anglimp-South Waghi	Kugmar	Highlands	This study - new collection	1	Q1a	[illegible]
PNG113	Papua New Guinea	Chimbu	Gumine	ND	Highlands	This study - new collection	1	Q1a1	[illegible]
PNG116	Papua New Guinea	Western Highlands	Mount Hagen	ND	Highlands	This study - new collection	1	M29a	[illegible]
PNG117	Papua New Guinea	East New Britain	Gazelle	ND	Near Oceania	This study - new collection	1	Q1	[illegible]
PNG122	Papua New Guinea	East Sepik	Angoram	Kundiman	Coastal/Lowland	This study - new collection	1	Q1a1a	[illegible]
PNG123	Papua New Guinea	East Sepik	Angoram	Imboi	Coastal/Lowland	This study - new collection	1	Q1	[illegible]
PNG124	Papua New Guinea	East Sepik	Angoram	Auwin	Coastal/Lowland	This study - new collection	1	Q1a1a	[illegible]
PNG125	Papua New Guinea	East Sepik	Angoram	Auwin	Coastal/Lowland	This study - new collection	1	Q1	A73G T89C G92A T146C A263G T489C A750G A1438G A2706G T4117C A4769G G5460A C7028T T7084C A8701G G8790A A8860G C8964T T9540C A10398G C10400T T10873C G11719A C12705T G12940A T13500C T14025C C14766T T14783C G15043A T15289C G15301A A15326G G15731A T15908C G16129A T16144C C16148T C16223T C16261T A16265C T16311C A16343G
PNG127	Papua New Guinea	East Sepik	Angoram	Auwin	Coastal/Lowland	This study - new collection	1	Q1a1a	A73G T89C G92A C96T T146C T152C T208C A263G T489C A750G C1009T A1438G A2706G T4117C A4769G G5460A A5843G G6017A C7028T C7681T A8701G G8790A A8860G C8964T G9266A T9540C A10398G C10400T T10873C G11719A G12406A T12414C C12705T G12940A T13500C T14025C C14766T T14783C G15043A G15301A G15317A A15326G G16129A T16144C [illegible]
PNG128	Papua New Guinea	East Sepik	Angoram	Ombromas	Coastal/Lowland	This study - new collection	1	Q1a1	A73G T89C T146C T208C A263G C309CCT T310C T489C A750G A1438G A2706G T4117C A4769G G5460A A5843G C7028T C7681T A8701G G8790A A8860G C8964T T9540C T9797C A10398G C10400T T10873C T11353C G11719A C12705T G12940A T13500C T14025C C14766T T14783C G15043A G15301A A15326G T16093C G16129A T16144C C16148T C16223T A16241G
PNG129	Papua New Guinea	East Sepik	Angoram	Auwin	Coastal/Lowland	This study - new collection	1	Q1	A73G T89C G92A T146C A263G C315CC T489C A750G A1438G A2706G T4117C A4769G G5460A C7028T T7084C A8701G G8790A A8860G C8964T T9540C A10398G C10400T T10873C G11719A C12705T G12940A T13500C T14025C C14766T T14783C G15043A T15289C G15301A A15326G G15731A T15908C G16129A T16144C C16148T C16223T C16261T A16265C T16311C [illegible]
PNG130	Papua New Guinea	East Sepik	Angoram	Auwin	Coastal/Lowland	This study - new collection	1	Q1	A73G T89C T146C G207A A263G T489C A750G A1438G A2706G G3483A T4117C T4500C A4769G G5460A A5843G G6261A C7028T A8701G G8790A A8860G C8964T T9540C G9554A A10398G C10400T T10873C G11719A C12705T G12940A T13500C T14025C C14766T T14783C G15043A G15301A A15326G G16129A T16144C C16148T C16192T C16223T A16265C T16311C
PNG131	Papua New Guinea	East Sepik	Angoram	Auwin	Coastal/Lowland	This study - new collection	1	Q1	A73G T89C G92A T146C A263G T489C A750G A1438G A2706G T4117C A4769G G5460A C7028T T8286C A8701G G8790A A8860G C8964T T9540C A10398G C10400T T10873C G11719A C12705T G12940A T13500C T14025C C14766T T14783C G15043A T15289C G15301A A15326G G15731A T15908C G16129A T16144C C16148T C16223T C16261T A16265C T16311C A16343G
PNG133	Papua New Guinea	East Sepik	Angoram	Kundiman	Coastal/Lowland	This study - new collection	1	Q1	A73G T89C G92A T146C T195C A263G T489C A750G T1420C A1438G G1719A A2706G T4117C T4500C A4769G T5201C G5460A A5843G C7028T A8701G G8790A A8860G C8964T G9225C T9540C A10398G C10400T T10873C G11719A C12705T G12940A T13500C T14025C C14766T T14783C G15043A G15301A A15326G G16129A T16144C C16148T C16223T A16241G A16265C [illegible]

* ND : Not determined

** Geographic affiliation of the samples was based on the location of the maternal grand-mother for the new samples and from the sampling location of the participants for the IMR collection.

*** Mitogenomes 1-obtained following Kircher et al 2012 and Arias et al 2017; 2-extracted from (unpublished) whole genomes sequences performed at the Centre National de Recherche en Génomique Humaine (CNRGH, France)
